# Venetoclax combined with low dose cytarabine compared to standard of care intensive chemotherapy for the treatment of favourable risk adult acute myeloid leukaemia (VICTOR): Study protocol for an international, open-label, multicentre, molecularly-guided randomised, phase II trial

**DOI:** 10.1186/s12885-022-10221-2

**Published:** 2022-11-14

**Authors:** Richard Dillon, Shanna Maycock, Aimee Jackson, Sonia Fox, Sylvie Freeman, Charles Craddock, Catherine Thomas, Emma Homer, Jane Leahy, Anna Mamwell, Nicola Potter, Nigel Russell, Andrew Wei, Hans Beier Ommen, Claire Hemmaway, Steve Knapper, Lucinda Billingham

**Affiliations:** 1 Department of Medical and Molecular Genetics, King’s College London, London, UK; 2 Department of Clinical Haematology, Guy’s and St Thomas’ National Health Service (NHS) Foundation Trust, London, UK; 3 Cancer Research UK Clinical Trials Unit (CRCTU), University of Birmingham, Edgbaston, Birmingham, B15 2TT UK; 4 Clinical Immunology Service, Institute of Immunology and Immunotherapy, University of Birmingham, Edgbaston, Birmingham, B15 2TT UK; 5 Centre for Clinical Haematology, Queen Elizabeth Hospital, University Hospitals Birmingham NHS Foundation Trust, Birmingham, UK; 6 Blood Cancer UK, London, UK; 7 School of Medicine, Clinical Sciences Building, The University of Nottingham, Nottingham City Hospital, Hucknall Road, Nottingham, NG5 1PB UK; 8 Peter MacCallum Cancer Centre, 305 Grattan Street, Melbourne. VIC 3000, Australia; 9 Department of Hematology, Aarhus University Hospital, Palle Juul-Jensens Boulevard 99, 8200 Aarhus C, Denmark; 10 Department of Haematology, Auckland City Hospital, Auckland, New Zealand; 11 Institute of Cancer and Genetics, School of Medicine, Cardiff University, Cardiff, CF14 4XN UK

**Keywords:** Clinical trial, Acute myeloid leukaemia, Bayesian non-inferiority design, Adaptive design, Venetoclax, Low-dose cytarabine

## Abstract

**Background:**

For patients with acute myeloid leukaemia (AML), the only potentially curative treatment is intensive chemotherapy (IC). This is highly toxic, particularly for patients > 60 years, potentially leading to prolonged hospitalisations requiring intensive supportive care, and sometimes treatment-related death. This also results in extensive healthcare costs and negatively impacts quality of life (QoL). Venetoclax with low-dose cytarabine (VEN + LDAC) is a novel, low-intensity treatment for AML patients who cannot receive IC. VEN + LDAC is given as an outpatient and toxicity appears significantly lower than with IC. Analysis of clinical trials performed to date are promising for patients with the genotype *NPM1*^mut^*FLT3* ITD^neg^, where remission and survival rates appear comparable to those achieved with IC.

**Methods:**

VICTOR is an international, two-arm, open-label, multi-centre, non-inferiority, randomised-controlled phase II trial to assess VEN + LDAC compared to standard of care (IC) as first-line treatment in older patients (initially aged ≥ 60 years) with newly diagnosed AML. The trial will recruit patients with a *NPM1*^mut^*FLT3* ITD^neg^ genotype; those with a favourable risk in relation to the experimental treatment. University of Birmingham is the UK co-ordinating centre, with national hubs in Aarhus University Hospital, Denmark, and Auckland District Health Board, New Zealand. The primary outcome is molecular event-free survival time where an event is defined as failure to achieve morphological complete response (CR) or CR with incomplete blood count recovery after two cycles of therapy; molecular persistence, progression or relapse requiring treatment change; morphological relapse, or; death. Secondary outcomes include cumulative resource use at 12- and 24-months, and QoL as assessed by EORTCQLQ-C30 and EQ-5D-3L at 3-, 6-, 12-, 18- and 24-months. The trial employs an innovative Bayesian design with target sample size of 156 patients aged > 60 years.

**Discussion:**

The principle underpinning the VICTOR trial is that the chance of cure for patients in the experimental arm should not be compromised, therefore, an adaptive design with regular checks on accumulating data has been employed, which will allow for a staged expansion of the trial population to include younger patients if, and when, there is sufficient evidence of non-inferiority in older patients.

**Trial registration:**

EudraCT: 2020–000,273-24; 21-Aug-2020.

ISRCTN: 15,567,173; 08-Dec-2020.

**Supplementary Information:**

The online version contains supplementary material available at 10.1186/s12885-022-10221-2.

## Background

Acute myeloid leukaemia (AML) is an aggressive haematological malignancy, which despite improvements to treatment and supportive care, remains fatal in the majority of patients. The only potentially curative treatment is intensive (or induction) chemotherapy (IC) followed in selected cases by allogeneic stem cell transplantation (SCT) where indicated and when a suitable donor is identified [1, 2]. IC is successful in inducing complete remission (CR) in 60–80% of younger patients (aged 16–60 years) [3, 4] and in around 50% of older patients ≥ 60 years [5, 6]. IC is highly toxic, due mainly to prolonged myelosuppression, which results in a high risk of severe bacterial and/or fungal infection. This is the most frequent cause of induction death, which occurs in 5–10% of patients aged < 60 years [3, 4, 7] and 10–20% of older individuals [7, 8]. Patients undergoing IC are usually hospitalised for a prolonged period and require intensive support with blood products and anti-infective agents, resulting in significant economic cost (estimated a £100,000-£400,000 per patient) and impact on quality of life (QoL) [9–14]. Additionally, patients treated with IC are at risk for several serious long-term side effects including cardiac and neurological sequelae, infertility and secondary cancers [15].

As the risk of mortality with IC increases with age and comorbidity [7, 8] older patients and those with severe pre-existing conditions are typically treated with non-intensive chemotherapy with either low-dose cytarabine (LDAC) or a hypomethylating agent (HMA, i.e., 5-azacitidine or decitabine) [1, 2]. Such treatments, however, display at best modest efficacy resulting in CR in only 10–30% of patients and median overall survival rates of approximately 6–12 months [16–19]. The B-cell lymphoma-2 (BCL2) inhibitor venetoclax (VEN), has been used in combination with LDAC or a HMA in patients not eligible to receive IC. These VEN-based combinations have been shown to produce CR rates between 50–75%, representing a major advance in the treatment of AML [20–22] and leading to the approval of VEN + HMA and VEN + LDAC combination therapy for patients with AML who are ineligible for IC in many countries including the UK [23].

Response to VEN based regimens in AML varies according to the molecular subgroup [24, 25]. The most impressive responses to date have been observed for patients harbouring mutations in *NPM1*, which is the most commonly mutated gene in AML accounting for approximately one third of cases. In this group, the combined phase II data (*n* = 27) demonstrate an overall response rate of 93% and two-year overall survival rate of 75% [20–22, 24, 25], moreover it appears that those *NPM1* mutated patients who do not respond to or who relapse shortly after treatment with VEN based combination therapy usually harbour the *FLT3* internal tandem duplication (ITD) [24].

The rate and durability of response to VEN based combinations in single arm studies for patients with *NPM1* mutated AML compares favourably with outcomes of patients treated with IC, for example adults with the favourable risk genotype *NPM1*^mut^ *FLT3* ITD^neg^ treated with IC in the intensive therapy arm of UK NCRI AML16 (median age 67 years, range 60–84 years) demonstrated overall survival rates at 2 and 3 years of 50% and 35% [26]. These data raise the possibility that the novel non-intensive combination VEN + LDAC may be equivalent or superior to IC in terms of disease-free survival, treatment-related mortality and QoL for patients with *NPM1* mutated AML who currently receive intensive therapy.

The VICTOR trial will, therefore, compare VEN + LDAC to standard of care IC as first-line treatment in older patients (initially aged ≥ 60 years) with de-novo AML with the genotype *NPM1*^mut^ *FLT3* ITD^neg^.

## Design

### Study design

**Fig. 1:**
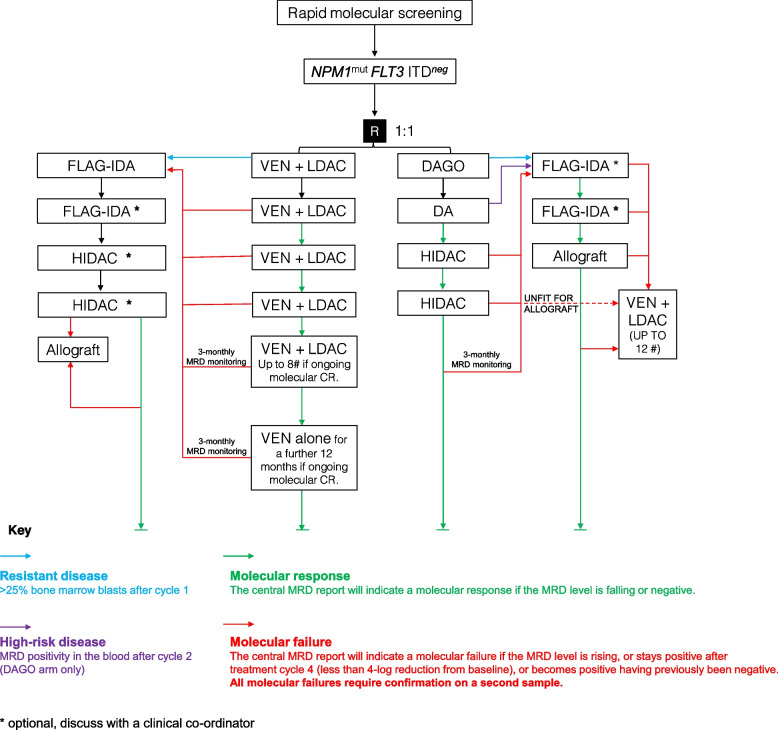
VICTOR trial schema VICTOR trial schema showing the patient pathway highlighting treatment decisions depending on disease status. #, cycles; CR, complete remission/response; DA, daunorubicin; DAGO, daunorubicin, cytarabine and gemtuzumab ozogamicin; FLAG-IDA, fludarabine, cytarabine and idarubicin; HIDAC, high-dose cytarabine; LDAC, low-dose cytarabine; MRD, minimal residual disease; R, randomisation; VEN, venetoclax

The VICTOR trial is an international, two-arm, multicentre, non-blinded, randomised-controlled phase II clinical trial of VEN + LDAC compared to standard of care IC (daunorubicin, cytarabine and gemtuzumab ozogamicin (DAGO)) in adult AML patients. VICTOR is adaptive trial allowing a staged expansion of the population to include younger patients as evidence emerges of non-inferiority in the older patients. The trial will recruit patients with the *NPM1*^*mut*^* FLT3* ITD^neg^ genotype (Fig. 1) as these have a favourable risk in relation to the experimental treatment. University of Birmingham is the UK co-ordinating centre, with national hubs in Aarhus University Hospital, Denmark, and Auckland District Health Board, New Zealand. The trial is designed to determine if there is sufficient evidence that VEN + LDAC is non-inferior and potentially beneficial compared to IC to warrant further research in a phase III trial. The trial has a Bayesian design with target sample size of 156 patients in the > 60 age group.

Randomisation will be 1:1 performed electronically using a computer program developed by the Cancer Research UK Clinical Trials Unit (CRCTU), Birmingham, UK. Stratification variables are:

- Age (60–69 years versus 70 + years (a further variable of 50–59 years to be included when/if required))
- Sex
- Performance status (0 versus 1 and 2)

This trial aims to recruit patients at participating centres following referral for suspected AML or at multi-disciplinary team meetings allowing referral to the trial site from other centres in the UK, Denmark, and New Zealand. Recruitment will be over two years, with participants followed up for at least two years. A list of the VICTOR trial centres can be requested from the VICTOR Trial Office (VICTOR@trials.bham.ac.uk). The Standard Protocol Items: Recommendations for Intervention Trials (SPIRIT) checklist is provided as Supplementary Appendix 1 [27]. The World Health Organization (WHO) Trial Registration Data Set is provided in Supplementary Appendix 2.

### Patient and public involvement

Two patient representatives, Jane Leahy and Anna Mamwell, co-developed the VICTOR Trial protocol by reviewing and refining the protocol, participant-facing documents and providing input into the statistical design of the study, particularly regarding selection of the pre-specified non-inferiority margin. As members of the trial management group (TMG) they will assess study conduct, will be involved in review of any amendments, and will support dissemination of the study results through existing advocacy activities and through social media channels.

### Patient selection

The key eligible criteria are listed in Table 1.

**Table 1:** Key patient eligibility criteria for the VICTOR trial

Inclusion Criteria
• Diagnosis of CD33 positive AML • Age ≥ 60 years (prior to the interim analyses performed after enrolment of 50 and 100 patients) • Genotype *NPM1*^mut^ *FLT3* ITD^neg^ (*FLT3*- Tyrosine Kinase Domain mutation, TKD, is permitted) • Considered fit for intensive chemotherapy with anthracyclines by treating physician • Eastern Cooperative Oncology Group (ECOG) performance status 0–2 • Serum creatinine ≤ 1.5 × ULN (upper limit of normal) • Serum alanine aminotransferase (ALT) and aspartate aminotransferase (AST) ≤ 2.5 ULN and bilirubin ≤ 2 × ULN • Able to provide written informed consent
**Exclusion Criteria**
• Previous chemotherapy for AML or any antecedent haematological condition, with the exception of hydroxycarbamide to control white blood cell count • Other active malignancy requiring treatment • Newly diagnosed or uncontrolled HIV or hepatitis B or C infection. Patients with known chronic infections may enrol if the last two tests for viral load have been negative and their current therapy does not include a protease inhibitor or a non-nucleoside reverse-transcriptase inhibitor • Pregnant and lactating patients (patients of childbearing potential must have a negative pregnancy test prior to study entry) • Females of childbearing potential, and their partners, not willing to use adequate contraception during and for up to 7 months after treatment • Patients who are unable to swallow tablets whole • Known hypersensitivity to any of the investigational medicinal products • Patients known to require vaccination with a live vaccine during the treatment period

*AML* acute myeloid leukaemia, *ULN* upper limit of normal

### Screening and consent

It is the responsibility of the investigator to obtain written informed consent for each patient prior to performing any trial related procedure in compliance with national regulations. Country specific patient information sheets (PIS) are provided to facilitate this process. The study has two PIS – one to allow collection of a blood sample to be sent for rapid molecular screening in order to confirm eligibility and the main trial PIS with full study information which should only be provided to the patient only once the genotype is identified as *NPM1*^mut^ *FLT3* ITD^neg^. Research nurses or specialist registrars are permitted to obtain informed consent for the blood sample collection and rapid molecular screening; however only Investigators may obtain consent for the main study. Supplementary Appendix 3 contains the exemplar VICTOR trial informed consent forms, with Supplementary Appendix 4 the PIS.

During screening, patients will be registered and a peripheral blood sample then sent to the trial laboratory at Guy’s Hospital (for patients in the UK and Denmark) or Auckland (for patients in New Zealand) for rapid molecular screening to test for the eligible genotype *NPM1*^*mut*^* FLT3* ITD^neg^. Patients will only be randomised if the eligible genotype is confirmed.

### Interventions

In both arms, cycles are up to 42 days in duration; this includes a 28-day treatment cycle with days 29–42 to allow for neutrophil and platelet count recovery. Patients will be assessed for response after each cycle of treatment once counts have recovered for the first 4 cycles, and subsequently every 3 cycles from cycle 6 onwards. The permitted dose schedules for both arms are described in Table 2.

**Table 2:** Permitted treatment schedules within the VICTOR trial

Drug	Dose	Method	Day of Cycle
Standard of Care (DAGO) Arm *			
**Cycle 1** (inpatient or ambulatory care setting) **			
Daunorubicin	60 mg/m^2^	1 h IV infusion	1, 3, 5
Cytarabine	100 mg/m^2^ (total 20 doses)	12-hourly by IV push	1–10
Gemtuzumab ozogamicin	3 mg/m^2^ (capped at 5 mg^#^)	2-h IV Infusion	1, 4 (or 4, 7)†
**Cycle 2** (inpatient or ambulatory care setting) **			
Daunorubicin	50 mg/m^2^	1 h IV infusion	1, 3, 5
Cytarabine	100 mg/m^2^ (total 16 doses)	12-hourly by IV push	1–8
**Cycles 3 and 4** (may be given as outpatient, ambulatory care setting or inpatient, as per local practice)			
Cytarabine			
< 60 years	3 g/m^2^ twice a day	12-hourly by IV push	1, 3, 5
60–69 years	1.5 g/m^2^ twice a day	12-hourly by IV push	1, 3, 5
≥ 70 years	1 g/m^2^ once daily	12-hourly by IV push	1–5
**Venetoclax and Low-dose Cytarabine Arm**			
**Cycle 1** (inpatient^α^)			
Cytarabine	20 mg/m^2^	Subcutaneous^β^	1–10
Venetoclax	100 mg	Oral	1
	200 mg	Oral	2
	400 mg	Oral	3
	100 mg	Oral	4–28
**Cycles 2–4** (outpatient (up to 12 cycles may be given))			
Cytarabine	20 mg/m^2^	Subcutaneous^β^	1–10
Venetoclax^γ^			
Either	100 mg	Oral	1–28
Or	600 mg	Oral	1–28
Accompanying supportive care (one of the following is mandated during all cycles):			
Aciclovir	400 mg twice a day	Oral	1–28
Valaciclovir	500 mg once a day	Oral	1–28
Posaconazole	300 mg twice a day	Oral	4
	300 mg once a day		5–28
Voriconazole	400 mg twice a day	Oral	4
	200 mg twice a day		5–28
Optional supportive care (one of the following is permitted):			
Ciprofloxacin	500 mg twice a day		1–28
Levofloxacin	500 mg once a day		1–28

^*^ May be administered as per local practice

^**^ If current standard of care is treatment in ambulatory care setting or outpatient day unit, this is permitted at the discretion of the treating investigator

^#^ Unless local standard practice is to dose cap at 4.5 mg

^†^ Gemtuzumab ozogamicin (GO) should be given on days 4 and 7 if white blood cell count ≥ 30 × 10^9^/L on day 1. For New Zealand sites standard of care will consistent of daunorubicin and cytarabine only until GO is available for use

^α^ Patients should generally be admitted to hospital for cycle 1 of treatment unless there are established local protocols for ambulatory management of induction therapy

^β^ Method of administration may be as per local practice, including self-administration

^γ^ 100 mg dose used if patient remains on anti-infective prophylaxis, 600 mg if patients stop anti-infective prophylaxis

Following cycle 1 the starting criteria for each cycle (cycle 2–4 of DAGO and cycle 2–12 of VEN + LDAC) are a neutrophil count > 1.0 × 10^9^/L and a platelet count > 75 × 10^9^/L. The subsequent cycle may be delayed by a further two weeks until the counts recover, however, if not recovered six weeks after the start of the preceding cycle (i.e., by day 42), a bone marrow aspirate and trephine will be performed to confirm a morphological leukaemia free state (MLFS). If confirmed, and in the absence of molecular progression, a further delay in treatment may be recommended after discussion with the clinical co-ordinators. If a MLFS is not confirmed, patients may receive salvage chemotherapy.

The FLAG-Ida regimen is recommended for salvage therapy as detailed below and adjusted according to the patient’s age as follows (although the final decision on salvage regimen will be made by investigators, taking into account performance status and other co-morbidities).

- Age < 60: Fludarabine 30 mg/m^2^ (D1-5), cytarabine 2000 mg/m^2^ (D1-5) and idarubicin 8 mg/m.^2^ (D3-5)
- Age 60–69: Fludarabine 30 mg/m^2^ (D1-5), cytarabine 1000 mg/m^2^ (D1-5) and idarubicin 8 mg/m.^2^ (D3-5)
- Age ≥ 70: Fludarabine 25 mg/m^2^ (D1-5), cytarabine 1000 mg/m^2^ (D1-5) and idarubicin 5 mg/m.^2^ (D3-5)

Granulocyte-colony stimulating factor is given daily on days 1–5 and then from day 10 until neutrophil count recovery (> 1.0 × 10^9^/L) for all patients. Supportive care should be given according to local guidelines for this regimen which should include prophylaxis for *Pneumocystis pneumonia*.

In the following circumstances, the VICTOR trial allows for patients randomised to receive standard of care (DAGO) to crossover to receive VEN + LDAC treatment for up to 12 cycles:

- Any patient who meets criteria to receive salvage chemotherapy, but is judged by the investigator to be unfit to receive this for any reason.
- Any patient who has received salvage chemotherapy and meets criteria to receive a transplant but cannot receive this because of comorbidity, performance status or donor availability.
- Any patient who has received salvage chemotherapy but who experiences molecular progression.
- Any patient who has received a transplant and experiences molecular persistence, molecular progression or molecular relapse.

Patients who crossover will be followed up for all trial outcome measures; the occurrence of molecular complete remission after crossover, molecular event-free survival time and overall survival time from crossover will be reported descriptively in this single cohort of patients. The VICTOR schedule of events has been included in Supplementary Appendix 5.

### Treatment compliance

The local trial pharmacist will be responsible for maintaining and updating the drug accountability logs in the Pharmacy File for DAGO, venetoclax, and cytarabine throughout the study, which will be used to monitor compliance. Upon treatment discontinuation patients will be asked to return all unfinished bottles of venetoclax to the site research team for reconciliation.

### Dose modifications and discontinuations

Recommended dose modifications are listed in Table 3.

**Table 3:** Recommended dose modifications within the VICTOR trial

Standard of Care (DAGO) Arm	
**Observation**	**Recommended Modification**
Serum creatinine (mmol/L)	Daunorubicin dose
< 105	100%
105–265	75%
> 265	50%
Bilirubin > 2 × ULN and AST/ALT > 2.5 × ULN	Postpone gemtuzumab ozogamicin dose until less than these levels. Consider omitting the scheduled dose if delayed more than 2 days between sequential infusions
Bilirubin (μmol/L)	Daunorubicin dose
< 20	100%
20–50	75%
> 50	50%
Bilirubin (μmol/L)	Cytarabine dose
> 34	50%
**Venetoclax and Low-dose Cytarabine Arm**	
During venetoclax maintenance:	
Upon reoccurrence of grade 3 or 4 non-haematological toxicity, or grade 4 haematological toxicities, except lymphopenia	
**Dose at interruption (mg)**	Restart dose (mg*)
400	300
300	200
200	100
100	50**

^*^ The modified dose should be continued for 1 week before increasing the dose

^**^ As the venetoclax is only available in 100 mg strength, if a dose reduction to 50 mg is required, the patient should take 100 mg every other day

*ALT* alanine aminotransferase, *AST* aspartate transaminase, *ULN* upper limit of normal

If signs and symptoms of veno-occlusive disease or sinusoidal obstruction syndrome occur in patients treated with gemtuzumab ozogamicin (GO), defibrotide treatment can be considered and GO should be discontinued.

For patients receiving VEN and experiencing grade 3–4 abnormal liver function tests VEN and any potentially hepatotoxic drugs (including azole antifungals) should be withheld until these have resolved to grade 2 or below. Venetoclax (and the azole antifungal if applicable) should be restarted at the original dose. During maintenance, venetoclax may be withheld for any grade 3 or 4 non-haematological toxicity, or grade 4 haematological toxicities (except lymphopenia). Once the toxicity has resolved to grade 1 or baseline level (recovery), therapy with VEN may be restarted at the same dose. If the toxicity recurs, and for any subsequent occurrences, the dose reduction guidelines in Table 3. For patients who require dose reductions to less than 100 mg for more than 2 weeks, discontinuation of VEN should be considered.

### Concomitant medication

Strong or moderate CYP3A inhibitors and inducers, and P-gp and BCRP inhibitors should be avoided in patients receiving venetoclax where possible. The administration of live vaccines is prohibited for patients on either arm for the duration of treatment and thereafter until B-cell recovery.

### Trial outcomes

The primary outcome is molecular event-free survival time (mEFS) where an event is defined as follows:

- Failure to achieve morphological CR or CR with incomplete blood count recovery (CRi) after two cycles of therapy;
- Molecular persistence, progression or relapse requiring treatment change;
- Morphological relapse, or;
- Death.

Morphological CR is defined as < 5% blasts in a cellular bone marrow with neutrophil count ≥ 1 × 10^9^/L and platelet count ≥ 100 × 10^9^L, and CRi as < 5% blasts but with neutropenia (neutrophil count < 1 × 10^9^/L) or thrombocytopenia (platelet count < 100 × 10^9^/L) [28].

Molecular persistence is defined as detectable *NPM1* mutant transcripts present after completion of treatment with less than a 4 log_10_ reduction from baseline in the bone marrow, confirmed on a second consecutive sample; molecular progression an increase in *NPM1* mutant transcript levels by > 1 log_10_, confirmed on a second sample, and molecular relapse as detectable *NPM1* mutant transcripts, confirmed on a second consecutive sample showing an increase of > 1 log_10_ in a patient who previously tested negative in at least one technically adequate bone marrow sample (i.e., *ABL* C_t_ < 26.5 or *ABL* copy number > 10,000) [29].

Morphological relapse is defined as ≥ 5% blasts in the blood or bone marrow in a patient with a previously documented CR, CRi or morphological leukaemia free state (MLFS: < 5% bone marrow blasts, no auer rods or extramedullary disease, does not meet criteria for CR or CRi (i.e., blood count recovery is below the specified levels) [28].

Secondary outcomes include: Occurrence of morphological complete remission (CR or CRi) by the end of the second cycle of treatment; death within 30 and 60 days from trial entry; overall survival time (OS) from date of randomisation; time to morphological relapse from date of morphological complete remission; time to molecular relapse from date of molecular complete remission; cumulative occurrence of grade 3 and 4 adverse events at 12 and 24 months; prevalence of molecular complete remission at month 3, 6 and 12; cumulative resource use at 12 and 24 months including hospital admission days, blood product usage and days on intravenous antibiotics and antifungals; health-related quality of life at month 3, 6, 12, 18 and 24; change in performance status from baseline at month 3, 6, 12, 18 and 24, and; change in Comprehensive Geriatric Assessment (CGA) [30] from baseline at month 12 and 24.

Quality of life questionnaires (EORTC QLQ 30 [31] and EQ-5D-3L [32]) will be completed independently by patients.

### Statistical analysis plan

The aim of the statistical analysis is to determine if there is sufficient evidence that VEN + LDAC is non-inferior to IC in terms of mEFS to warrant further research in a phase III trial and in addition that VEN + LDAC has greater benefit than IC in terms of secondary outcomes. As a non-inferiority trial, statistical analysis will be carried out on a per protocol basis which includes all patients who have received the first dose of protocol treatment. Patients who are randomised but do not receive any treatment will be replaced. Analysis will primarily focus on the ≥ 60 age group, if younger patients are recruited following the interim analysis they will be analysed as a parallel cohort.

Outcome measures will be analysed under a Bayesian framework using conjugate models with minimally informative prior distributions. For all time-to-event outcomes, the Kaplan–Meier method will be used to plot the data. The posterior probability distribution of the true log hazard ratio will be estimated using a Normal-Normal conjugate model and the hazard ratio for comparison of the treatment arms will be estimated from the exponential of the mean of this distribution together with 80% and 95% credible intervals. For the primary outcome measure of mEFS, the posterior probability that the hazard ratio (HR) is less than the non-inferiority margin of 1.29 will be reported and enable decision-making (see next section for justification of non-inferiority margin). The decision criteria for both the interim analysis and final analysis are based on there being at least 80% chance that VEN + LDAC is truly non-inferior. Thus, if the posterior probability distribution estimates that the probability of mEFS HR < 1.29 is at least 80% given the observed data and a minimally informative prior, this would be taken as a “go” decision.

For all binary outcomes that are recording the occurrence of an event, the posterior probability distributions will be estimated for the true proportion of events using a beta-binomial conjugate model and the difference in proportions will estimated together with 80% and 95% credible intervals.

Each of the eight measures contributing to cumulative resource use will be summarised individually. In addition, each resource use will be combined with unit costs and summed to give a total cost per patient. The mean total cost will be calculated for each treatment arm and the absolute difference estimated.

Quality of life, change in performance status and change in CGA will be summarised and displayed graphically.

### Sample size determination

VICTOR uses a Bayesian approach to estimation and decision-making, based on the design proposed by Neuenschwander and colleagues [33]. The one-year mEFS rate on DAGO is expected to be 63% based on molecular monitoring and clinical outcome data from patients aged ≥ 60y with the genotype *NPM1*^mut^ *FLT3* ITD^neg^ treated with IC (Unpublished observations, R. Dillon). A decrease of this one-year mEFS rate on VEN + LDAC to 55% would be acceptable given the benefits of VEN + LDAC in comparison to DAGO, this equates to a non-inferiority margin in terms of hazard ratios of 1.29. Further to this, there is potential for the VEN + LDAC arm to actually be superior to DAGO in terms of mEFS. Phase II data (*n* = 27) gave a 2-year overall survival rate of 75% with VEN [34], which could be extrapolated back to suggest that a 1-year mEFS rate of 75% may be a realistic outcome which equates to a superiority HR of 0.62.

As a phase II proof of concept trial that is aiming for a moderate sample size, it is recognised that a more liberal type I and type II errors are acceptable. We have selected a one-sided alpha-level of 20% that then defines our decision criteria at the final analysis. We have also specified an additional criterion for the sample size to ensure that when the observed final estimate for the mEFS HR attains a required critical value of 1.1 then statistical significance will be achieved, i.e., this is the maximum observed values that would generate a “go” decision under the Bayesian framework specified above. A critical value marginally greater than 1 was chosen because of the potential substantial patient benefits of VEN + LDAC over DAGO. Statistical simulations show that 116 events are required to achieve the criteria specified above. In addition, with this number of events, the design has an 80% probability to correctly conclude non-inferiority when the true HR is 0.94. With a 2-year recruitment period, a 2-year follow-up period and expected one-year mEFS rate of 63%, we predict that the trial needs to recruit a total of 156 patients in order to observe the 116 events required for the trial.

The sample size calculation is based on recruitment of patients aged ≥ 60 years. If non-inferiority of VEN + LDAC is demonstrated, younger patients aged 50–59 years may be recruited in a parallel cohort. It is estimated that an additional 30–50 patients will be recruited in the younger cohort to allow an evaluation against the older cohort to determine if the trend in effect is comparable. There is no formal statistically-based sample size calculations for this additional cohort and no set minimum/maximum number of patients.

The trial is adaptive and will allow a staged expansion of the population to include younger patients as evidence emerges of non-inferiority in the older patients. This decision will first be considered at the interim analysis when 50 patients have been recruited and have sufficient follow up. Further evaluation can take place at later time points if the DMC require further certainty.

### Tissue and blood samples

The blood and bone marrow sampling schedule reflects UK standard of care practice as far as possible, so that trial patients will not be exposed to a greater number of sampling procedures than those treated off-study. A bone marrow aspirate and trephine sample will be taken during screening; samples will be taken for local morphology, histopathology, cytogenetics and flow cytometry and in addition an aspirate sample will be sent to the trial laboratory. Aspirates will then be taken at the end of each cycle for cycles 1–4 (once cell counts have recovered) to determine morphological response assessment; an aspirate sample will also be sent to Guy’s Hospital at each time point for molecular response assessment. A trephine will only be required during follow up if cell counts have not recovered by day 42 of the cycle, to confirm a MLFS. Following the first four cycles of treatment, a bone marrow aspirate will be performed and at month six (which will usually be the post-cycle 4 time point), and then every three months for two years from trial entry, unless the post cycle four bone marrow aspirate tests minimal residual disease (MRD) positive with a reduction of less than 4 log_10_ from baseline and this is confirmed on a second sample. In this case molecular persistence is diagnosed and patients should change treatment, as outlined above. If the sample is MRD positive but the reduction is more than 4 log_10_ from baseline, bone marrow aspirates should be performed every 4–6 weeks until the patient enters molecular complete remission, experiences molecular failure, or the levels have been stable for > 3 months, when the frequency of monitoring may be reduced to every three months.

A peripheral blood sample will be collected at diagnosis to determine eligibility, this will be sent fresh to the trial laboratory for rapid screening for *FLT3* and *NPM1* mutations, which is performed and reported within 24 h of sample receipt. Following trial entry, paired peripheral blood samples will be taken at the same time as bone marrow aspirates i.e., baseline (an additional pre-treatment blood sample is required as a baseline sample), after each cycle of treatment for four cycles, then at month six (which will be the post cycle 4 sample for most patients), then three-monthly for two years from trial entry. These samples will be shipped fresh for patients in the UK and Denmark together with the paired bone marrow aspirate. For patients in New Zealand, both peripheral blood and bone marrow samples will be processed to cDNA in Auckland and then sent to London.

All samples will be collected in accordance with national regulations and requirements including standard operating procedures for logistics and infrastructure. Samples will be taken in appropriately licensed premises and transported in accordance with the Human Tissue Authority guidelines and NHS trust policies.

### Adverse events reporting and analysis

The collection and reporting of adverse events (AEs) as measured by National Cancer Institute (NCI) Common Terminology Criteria for Adverse Events (CTCAE), version 4.0 [35], will be in accordance with the Research Governance Framework for Health and Social Care and the requirements of the National Research Ethics Service. Definitions of different types of AEs are listed in online Supplementary Appendix 6. The reporting period for AEs will be between the date of commencement of protocol defined treatment for up to 28 days after the last dose of investigational medicinal product (IMP). The investigator should assess the seriousness and causality (relatedness) of all AEs experienced by the patient (this should be documented in the source data) with reference to the protocol. All grade 3 and above medical occurrences which meet the definition of an AE should be reported, with the exception of abnormal laboratory findings which should only be reported if the event:

- Results in the early discontinuation of trial treatment, and/or;
- Requires a dose modification or interruption or any other therapeutic intervention or is judged to be of significant clinical importance.

If a laboratory abnormality is one component of a diagnosis or syndrome, then only the diagnosis or syndrome should be recorded as an AE. Pre-existing conditions should only be reported if the condition worsens by at least one CTCAE grade. All AEs will be reported using the applicable electronic case report form (eCRF).

The following events should not be reported as serious adverse events (SAEs):

Hospitalisations for:

- Protocol defined treatment
- Pre-planned elective procedures unless the condition worsens
- Treatment for progression of the patient’s cancer

Progression or death as a result of the patient’s cancer (unless the investigator considers it related to study treatment), as this information is captured elsewhere on the Case Report Form.

Development of neutropenia (< 1.0 × 10^9^/L) or thrombocytopenia (< 50 × 10^9^/L) within 42 days of the start of the treatment cycle.

Events that do not require expedited (immediate) reporting which will be reported on an expected serious adverse reaction (SAR) form.

Patients receiving chemotherapy may require admission to hospital for appropriate medical intervention following development of some of the more severe known side effects of treatment. For this reason, infection-based SAEs do not require expedited (immediate) reporting by site and are not regarded as unexpected for the purpose of this trial, unless the event proves fatal or requires admission to a high dependency or intensive care facility. An expected SAR form can be completed for these specific events instead of an SAE form.

One of the main adverse events associated with the use of VEN is tumour lysis syndrome. This syndrome occurs when an effective treatment causes a large number of cancer cells to die quickly, resulting in electrolyte and metabolic abnormalities which can cause nausea, seizures, kidney damage, cardiac toxicities and death. Tumour lysis syndrome is largely seen in chronic lymphoblastic leukaemia patients treated with VEN; with studies in AML have not demonstrated TLS to be an adverse event of concern in this population [20–22]. However, tumour lysis syndrome will be included as an adverse event of special interest during VICTOR and should be reported as a SAE.

### Data management

Case report forms (CRF) can be entered online at https://www.cancertrials.bham.ac.uk/victorlive. Authorised staff at sites will require an individual secure login username and password to access this online data entry system. For the purposes of this trial the QoL questionnaires will be captured on paper and entered onto the eRDC system by the VICTOR Trial Office. Data reported on each CRF should be consistent with the source data or the discrepancies should be explained. If information is not known, this must be clearly indicated on the CRF. All missing and ambiguous data will be queried. All sections are to be completed.

All trial records must be archived and securely retained for at least 25 years. No documents will be destroyed without prior approval from the sponsor, via the central VICTOR Trial Office. On-site monitoring will be carried out as required following a risk assessment and as documented in the Quality Management Plan. Any monitoring activities will be reported to the central VICTOR Trial Office and any issues noted will be followed up to resolution. VICTOR will also be centrally monitored, which may trigger additional on-site monitoring.

The CRCTU will hold the final trial dataset and will be responsible for the controlled sharing of anonymised clinical trial data with the wider research community to maximise potential patient benefit while protecting the privacy and confidentiality of trial participants. Data anonymised in compliance with the Information Commissioners Office requirements, using a procedure based on guidelines from the Medical Research Council (MRC) Methodology Hubs, will be available for sharing with researchers outside of the trials team within 12 months of the primary publication.

### Trial organisation structure

The University of Birmingham will act as single sponsor for this multi-centre study: Support Group, Aston Webb Building, Room 119, Birmingham, B15 2TT. Email: researchgovernance@contacts.bham.ac.uk. The trial is being conducted under the auspices of the CRCTU, University of Birmingham according to their local procedures. The TMG will be responsible for the day-to-day running and management of the trial. Members of the TMG include the Chief Investigator, University of Birmingham lead investigator, co-investigators, patient representatives, sub-study specialists, the trial management team leader (or delegate), the trial biostatistician, trial coordinator, and monitor. The TMG will have regular meetings during recruitment.

An independent trial steering committee (TSC) will be set up to oversee the conduct of the trial. The TSC will be led by the independent Chair, Dr Christopher Hourigan, with membership including an independent clinician, Dr Lynn Quek, independent statistician (to be confirmed), a representative from the sponsor and at least one patient advocate. Selected members of the TMG including the Chief Investigator, trial biostatistician and co-Investigators will report to the TSC. The TSC will operate in accordance with a trial specific charter based upon the template created by the Damocles Group to supervise the conduct of the trial, monitoring progress including recruitment, data completeness, losses to follow-up, and deviations from the protocol. They will make recommendations about conduct and continuation of the trial to the sponsor. The TSC will meet shortly before commencement of the trial and then 6-monthly thereafter after the DMC meeting.

The DMC will consist of independent clinicians Prof Roland Walter and Prof Claire Harrison, as well as an independent statistician, Dr Amy Kirkwood. Data analyses will be supplied in confidence to the DMC, which will be asked to give advice on whether the accumulated data from the trial, together with the results from other relevant research, justifies the continuing recruitment of further patients. The DMC will meet every six months while patients are on treatment. Additional meetings may be called if recruitment is much faster than anticipated and the DMC may, at their discretion, request to meet more frequently. An emergency meeting may also be convened if a safety issue is identified. The DMC will report to the TMG who will convey the findings of the DMC to the TSC and the competent authority*.* The DMC may consider recommending the discontinuation of the trial if the recruitment rate or data quality are unacceptable or if any issues are identified which may compromise patient safety. Based on interim analyses, the DMC will consider making a recommendation to expand the eligible population to include younger patients. The trial would also stop early if the interim analyses showed differences between treatments that were deemed to be convincing to the clinical community.

### Confidentiality

Confidential information collected during the trial will be stored in accordance with the General Data Protection Regulation (GDPR) 2018. As specified in the PIS and with the patients’ consent, patients will be identified using only their date of birth and unique trial ID number. Authorised staff may have access to the records for quality assurance and audit purposes. The Trials Office maintains the confidentiality of all patients’ data and will not disclose information by which patients may be identified to any third party other than those directly involved in the treatment of the patient and organisations for which the patient has given explicit consent for data transfer (e.g., laboratory staff).

### Dissemination of results and publication policy

A meeting will be held after the end of the study to allow discussion of the main results among the collaborators prior to publication. Results of the primary and secondary endpoints will be submitted for publication in peer-reviewed journals. Manuscripts will be prepared by the TMG and authorship determined by mutual agreement. A lay summary of the results will be published on the Cancer Research UK website.

### Trial status

Recruitment for the trial opened in June 2021 and recruitment is expected to last two years.

## Discussion

Non-inferiority trials in the phase II setting are rarely seen as it is perceived that they can only be done with large sample sizes, but the VICTOR trial has adopted the principle that it ‘will not deliver definitive evidence but can be considered as a sensible gate-keeper: a proof of concept for further development of the new treatment’ [33]. The trial design has been based on this principle.

The study design used in VICTOR is adaptive in terms of minimum age at trial entry. The maximum benefit of VEN + LDAC is expected to be seen in older adults who are at highest risk of complications from, and have the least favourable outcomes with IC. It is anticipated that any advantage of VEN + LDAC will be amplified in this age group, therefore, this study will initially enrol older adults (aged ≥ 60 years) considered fit for intensive therapy who would normally be assigned to IC. The adaptive design allows a staged expansion of the population to gradually include younger patients if and when there is sufficient evidence of non-inferiority in the older patients.

Regular checks on accumulating data reported to the DMC are incorporated in to the study design to ensure that the chance of cure for patients in the experimental arm is not compromised. Additional flexibility is also built into the treatment schedule to allow patients in both arms to cross over to the other treatment arm under certain circumstances. As well as opening up the possibility of an alternative treatment regime for patients who are unable to receive salvage chemotherapy or for whom salvage treatment has been ineffective, it is hoped that the flexibility of the VICTOR trial design provides patients with an attractive and patient-centred approach to their treatment, whilst maintaining the scientific rigour required to address the key outcomes of the trial.

If at the end of the trial there is enough evidence of non-inferiority the recommendation would be to continue to a phase III trial with a primary endpoint of overall survival. It is anticipated that this will be part of the AML20 trial but if that is not possible VICTOR has been designed to integrate into a phase III evaluation.

## Supplementary Information

**Additional file 1: Supplementary Appendix 1.** SPIRIT checklist for the VICTOR protocol A completed Standard Protocol Items: Recommendations for Intervention Trials (SPIRIT) checklist for the VICTOR protocol. **Supplementary Appendix 2.** WHO trial registration data set for the VICTOR trial The World Health Organization (WHO) trial registration data set for the VICTOR trial. **Supplementary Appendix 3.** VICTOR informed consent forms Exemplar informed consent and blood sample analysis consent form for the VICTOR trial. **Supplementary Appendix 4.** VICTOR patient information sheets Exemplar trial and blood sample analysis patient information sheets for VICTOR. **Supplementary Appendix 5.** VICTOR schedule of events Patient schedule of events for the VICTOR trial. **Supplementary Appendix 6.** Adverse event definitions Definitions of adverse events used for the VICTOR trial.

## Data Availability

Not applicable.

## Abbreviations

AE: Adverse event
AML: Acute myeloid leukaemia
BCL2: B-cell lymphoma-2
CGA: Comprehensive geriatric assessment
CR: Complete response
CRCTU: Cancer Research UK Clinical Trials Unit
CRF: Case report form
Cri: Complete response with incomplete blood count recovery
CTCAE: Common terminology criteria for adverse events
DAGO: Daunorubicin, cytarabine and gemtuzumab ozogamicin
DMC: Data monitoring committee
GO: Gemtuzumab ozogamicin
HMA: Hypomethylating agent
HR: Hazard ratios
IC: Intensive chemotherapy
ITD: Internal tandem duplication
LDAC: Low-dose cytarabine
mEFS: Molecular event-free survival time
MLFS: Morphological leukaemia free state
MRD: Minimal residual disease
OS: Overall survival time
PIS: Patient information sheet
QoL: Quality of life
SAE: Serious adverse event
SAR: Serious adverse reaction
TMG: Trial management group
TSC: Trial steering committee
VEN: Venetoclax
