# Supplementary material for: Venetoclax combined with low dose cytarabine compared to standard of care intensive chemotherapy for the treatment of favourable risk adult acute myeloid leukaemia (VICTOR): Study protocol for an international, open-label, multicentre, molecularly-guided randomised, phase II trial
Source: BMC Cancer. 2022 Nov 14;22:1174. doi: 10.1186/s12885-022-10221-2 (PMC9664612; doi:10.1186/s12885-022-10221-2)
Supplement: Supplementary file 1 — Additional file 1: Supplementary Appendix 1. SPIRIT checklist for the VICTOR protocol A completed Standard Protocol Items: Recommendations for Intervention Trials (SPIRIT) checklist for the VICTOR protocol. Supplementary Appendix 2. WHO trial registration data set for the VICTOR trial The World Health Organization (WHO) trial registration data set for the VICTOR trial. Supplementary Appendix 3. VICTOR informed consent forms Exemplar informed consent and blood sample analysis consent form for the VICTOR trial. Supplementary Appendix 4. VICTOR patient information sheets Exemplar trial and blood sample analysis patient information sheets for VICTOR. Supplementary Appendix 5. VICTOR schedule of events Patient schedule of events for the VICTOR trial. Supplementary Appendix 6. Adverse event definitions Definitions of adverse events used for the VICTOR trial. [file 12885_2022_10221_MOESM1_ESM.zip › VICTORprotocol_Appendix2-WHO v1.0R2.docx]

# Supplementary Appendix 2

| **Data category** | **Information** |
| --- | --- |
| Primary registry and trial identifying number | EudraCT Number 2020-000273-24 |
| Date of registration in primary registry | 21-Aug-2020 |
| Secondary identifying numbers | ISRCTN: 15567173 |
| Source(s) of monetary or material support | Cancer Research UK  AbbVie |
| Primary sponsor | University of Birmingham |
| Secondary sponsor(s) | n/a |
| Contact for public queries | Louise Hopkins: [l.m.dudley@bham.ac.uk](mailto:l.m.dudley@bham.ac.uk) |
| Contact for scientific queries | Louise Hopkins: [l.m.dudley@bham.ac.uk](mailto:l.m.dudley@bham.ac.uk) |
| Public title | A trial comparing the effectiveness and safety of venetoclax to standard chemotherapy in acute myeloid leukaemia patients |
| Scientific title | VICTOR: Venetoclax or Intensive Chemotherapy for Treatment Of Favourable Risk Acute Myeloid Leukaemia: a molecularly guided phase 2 study |
| Countries of recruitment | UK, Denmark, New Zealand |
| Health condition(s) or problem(s) studied | Newly diagnosed acute myeloid leukaemia |
| Intervention(s) | Venetoclax with low-dose cytarabine |
| Key inclusion and exclusion criteria: **Adult Group** | Ages eligible for study: Initially ≥60 years Sexes eligible for study: both Accepts healthy volunteers: no |
|  | Inclusion criteria: CD33 positive AML, genotype *NPM1*^mut^ *FLT3* ITD^neg^, performance status <2 |
|  | Exclusion criteria: Previous chemotherapy for AML, other active malignancy requiring treatment, pregnancy or breastfeeding patients |
| Study type | Interventional |
|  | Allocation: randomised, open-label |
|  | Primary purpose: Efficacy |
|  | Phase II |
| Date of first enrolment | 17-Aug-2021 |
| Target sample size | 156 |
| Recruitment status | Open |
| Primary outcome(s) | Molecular event-free survival time (mEFS) where an event is defined as:  • Failure to achieve morphological complete remission (CR) or CR with incomplete blood count recovery (CRi) after two cycles of therapy;  • Molecular persistence, progression or relapse requiring treatment change;  • Morphological relapse, or;  • Death  Response will be assessed after each cycle of treatment once counts have recovered for the first 4 cycles, and subsequently every 3 cycles from cycle 6 onwards. |
| Key secondary outcome(s) | Occurrence of morphological complete remission (CR or CRi) by the end of the second cycle of treatment  Death within 30 and 60 days from trial entry  Overall survival time from date of randomisation  Time to morphological relapse from date of morphological complete remission  Time to molecular relapse from date of molecular complete remission  Cumulative occurrence of grade 3 and 4 adverse events at 12 and 24 months  Prevalence of molecular complete remission at month 3, 6 and 12  Cumulative resource use at 12 and 24 months including hospital admission days, blood product usage and days on intravenous antibiotics and antifungals  Health-related quality of life at month 3, 6, 12, 18 and 24  Change in performance status from baseline at month 3, 6, 12, 18 and 24  Change in Comprehensive Geriatric Assessment from baseline at month 12 and 24 |
