# Supplementary material for: Venetoclax combined with low dose cytarabine compared to standard of care intensive chemotherapy for the treatment of favourable risk adult acute myeloid leukaemia (VICTOR): Study protocol for an international, open-label, multicentre, molecularly-guided randomised, phase II trial
Source: BMC Cancer. 2022 Nov 14;22:1174. doi: 10.1186/s12885-022-10221-2 (PMC9664612; doi:10.1186/s12885-022-10221-2)
Supplement: Supplementary file 1 — Additional file 1: Supplementary Appendix 1. SPIRIT checklist for the VICTOR protocol A completed Standard Protocol Items: Recommendations for Intervention Trials (SPIRIT) checklist for the VICTOR protocol. Supplementary Appendix 2. WHO trial registration data set for the VICTOR trial The World Health Organization (WHO) trial registration data set for the VICTOR trial. Supplementary Appendix 3. VICTOR informed consent forms Exemplar informed consent and blood sample analysis consent form for the VICTOR trial. Supplementary Appendix 4. VICTOR patient information sheets Exemplar trial and blood sample analysis patient information sheets for VICTOR. Supplementary Appendix 5. VICTOR schedule of events Patient schedule of events for the VICTOR trial. Supplementary Appendix 6. Adverse event definitions Definitions of adverse events used for the VICTOR trial. [file 12885_2022_10221_MOESM1_ESM.zip › VICTORprotocol_Appendix3 v1.0R2.docx]

*Print on hospital headed paper*


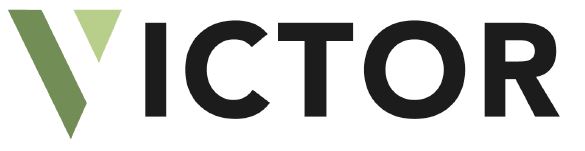


**V**enetoclax or **I**ntensive **C**hemotherapy for **T**reatment **O**f Favourable **R**isk Acute Myeloid Leukaemia: a molecularly guided phase 2 study

**INFORMED CONSENT FORM**

| **Site:** | **Patient Trial No. (TNO):** |
| --- | --- |
| **Principal Investigator:** | **EudraCT No.:** 2020-000273-24 |

### If you agree to take part in the VICTOR study, please:

- Initial each box
- Sign your full name at the end of this form

| **A. Taking part** | | |  |
| --- | --- | --- | --- |
| 1. I confirm that I have read and understand the VICTOR Patient Information Sheet, version 4.0, 6^th^ September 2021. I have had the opportunity to consider the information, ask questions and have had these answered to my satisfaction. | | |  |
| 1. I understand that my participation is voluntary and that I am free to withdraw at any time without giving any reason, without my medical care or legal rights being affected.   I understand that if I withdraw, some research may have already taken place using my samples and my data and that this research cannot be undone. | | |  |
| 1. I give permission for my date of birth and initials to be given to the Trial Office when I am randomised to the VICTOR trial, as well as a copy of this consent form to be sent to the Trial Office and laboratory at Guy’s Hospital*.* | | |  |
| 1. I agree to my GP being informed of my participation in this trial. | | |  |
| 1. I agree to take part in the VICTOR trial. | | |  |
| **B. Samples** | | |  |
| 1. I agree to donate as part of participation in the VICTOR study:    - Samples of blood    - Samples of my bone marrow | | |  |
| 1. I understand that my samples will be used for collecting DNA and analysis as described in the Patient Information Sheet. | | |  |
| 1. I agree to allow samples to be supplied to the laboratories at Guys Hospital, as outlined in the Patient Information Sheet for analysis (including the extraction, analysis and storage of my DNA).   **Optional -** The following is optional and will not affect entry into the trial, please initial for no or yes in the boxes:   1. I agree to allow my samples to be analysed at Guy’s hospital for the optional sub-study, as described in the Patient Information Sheet. 2. I consent to the storage of my samples remaining at the end of the study and their use in existing or future research which may involve genetic analysis. I understand that my samples or DNA from my samples could be shared with any UK or overseas research organisation. Research organisations may include academic institutions, clinical research groups or commercial (for-profit) companies. Any existing or future research carried out on my samples will have the relevant country specific approvals. I understand that future research may use new tests or techniques that are not yet known. | | | **No Yes** |
| **C. Data** | | |  |
| 1. I understand that relevant sections of my medical notes and data collected during the study may be looked at by individuals from the Trial Office, regulatory authorities, Sponsors, research collaborators, and/or NHS bodies, where it is relevant to my taking part in this research, safety monitoring, or licencing purposes. I give permission for these individuals to have access to my records. | | |  |
| 1. I understand that my data, and information from my samples will only be used by researchers in a form that protects my anonymity. Anonymised data and information derived from research samples may be shared with other research organisations in future research. This may include academic institutions, clinical research groups or commercial (for-profit) companies. I understand that this data may be transmitted outside the European Economic area to countries which may have a different level of data protection to that in the UK. | | |  |
| **Optional -** The following is optional and will not affect entry into the trial. This question is only relevant for patients in England who have agreed to allow their genome data to be added into the National Genomic Research Library please initial for no or yes in the boxes:   1. I give my permission for my NHS number and date of birth to be collected and used to link my medical data collected for the trial with the genome sequencing data and clinical data stored in the National Genomic Research Library, as described in the patient information sheet. | | | **No Yes** |
| **____________________________** | **_____________** | **_______________________________** | |
| **Name of participant** | **Date** | **Signature** | |
| **____________________________** | **_____________** | **_______________________________** | |
| **Name of person taking consent**  (You must have signed the Site Signature and Delegation Log) | **Date** | **Signature** | |

When completed, 1 for patient; 1 (original) for Investigator Site File; 1 to be kept in medical notes; 1 to be sent to CRCTU

# **Blood Sample Analysis at Guy’s Hospital Consent Form**

*Print on hospital headed paper*

| **Site:** | **Patient Initials:** |
| --- | --- |
| **Principal Investigator:** | **Patient Date of Birth:** |

**Please Initial Box**

1. I have read and understood the Patient Information Sheet – Blood Sample Analysis at Guy’s Hospital (Version 2.0, 07-Dec-2020).
2. I agree to provide blood samples (and a bone marrow sample if applicable) to be sent for analysis at Guy’s Hospital, London. I understand that my agreement is voluntary and that I am free to withdraw approval at any time without giving a reason and without my medical care and legal rights being affected.
3. I understand that the purpose of the analysis on the blood sample is to determine what genes are affected by my disease and therefore guide my treatment options.
4. I understand that if these samples provide the opportunity to enter the VICTOR study, separate information will be provided and I will be asked to sign a separate consent form.
5. I give permission for a copy of my consent form to be sent to the laboratory at Guy’s Hospital with the samples.

**Optional -** The following is optional and will not affect entry into the trial, please read the following two paragraphs and initial for no or yes in the boxes (if you select “no” your samples will be destroyed after the relevant tests have been completed):

If there are any left-over samples after the analysis has been performed, I consent to the

storage of my remaining samples and their use in existing or future research which may

involve genetic analysis. I understand that my samples or DNA from my samples could be

shared with any UK or overseas research organisation. Research organisations may include

academic institutions, clinical research groups or commercial (for-profit) companies. Any

existing or future research carried out on my samples will have the relevant country specific **No Yes**

approvals. I understand that future research may use new tests or techniques that are not

yet known.

I understand that my data, and information from my samples will only be used by

researchers in a form that protects my anonymity. Anonymised data and information

derived from research samples may be shared with other research organisations in future

research. This may include academic institutions, clinical research groups or

commercial (for-profit) companies. I understand that this data may be transmitted outside

the European Economic area to countries which may have a different level of data protection

to that in the UK.

______________________ ______________ ______________________

Name of patient Date Signature

______________________ ______________ ______________________

Name of person taking consent Date Signature

**When complete a copy to be given to the patient and the original to be kept in medical notes.**
