# Supplementary material for: Venetoclax combined with low dose cytarabine compared to standard of care intensive chemotherapy for the treatment of favourable risk adult acute myeloid leukaemia (VICTOR): Study protocol for an international, open-label, multicentre, molecularly-guided randomised, phase II trial
Source: BMC Cancer. 2022 Nov 14;22:1174. doi: 10.1186/s12885-022-10221-2 (PMC9664612; doi:10.1186/s12885-022-10221-2)
Supplement: Supplementary file 1 — Additional file 1: Supplementary Appendix 1. SPIRIT checklist for the VICTOR protocol A completed Standard Protocol Items: Recommendations for Intervention Trials (SPIRIT) checklist for the VICTOR protocol. Supplementary Appendix 2. WHO trial registration data set for the VICTOR trial The World Health Organization (WHO) trial registration data set for the VICTOR trial. Supplementary Appendix 3. VICTOR informed consent forms Exemplar informed consent and blood sample analysis consent form for the VICTOR trial. Supplementary Appendix 4. VICTOR patient information sheets Exemplar trial and blood sample analysis patient information sheets for VICTOR. Supplementary Appendix 5. VICTOR schedule of events Patient schedule of events for the VICTOR trial. Supplementary Appendix 6. Adverse event definitions Definitions of adverse events used for the VICTOR trial. [file 12885_2022_10221_MOESM1_ESM.zip › VICTORprotocol_Appendix4 v1.0R2.docx]

*Print on hospital headed paper*

**PATIENT INFORMATION SHEET**

*
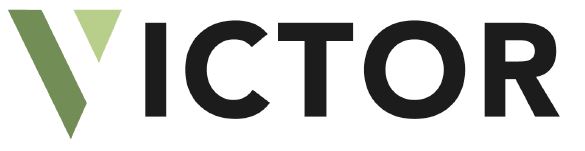
*

**V**enetoclax or **I**ntensive **C**hemotherapy for **T**reatment **O**f Favourable **R**isk Acute Myeloid Leukaemia: a molecularly guided phase 2 study

We would like to invite you to take part in a research study (also called a clinical trial) which is sponsored by the University of Birmingham. Joining the study is entirely up to you; before you decide we would like you to understand why the research is being done and what it would involve for you. Please take time to read this information carefully and discuss it with others if you wish.

Part 1 tells you the purpose of this study and what will happen to you if you take part, and part 2 gives you more detailed information about the conduct of the study. Do ask if anything is unclear, or if you would like more information. Take time to decide whether or not you wish to take part.

If you choose not to take part, this will not affect the care you get from your own doctors.

Thank you for reading this information sheet.

*________________________________________________________________________________________________________*

**Contents Page Page**

**Part 1**

What is the purpose of the VICTOR study? 2

Why have I been invited to take part? 3

How do I enter the study? 3

Do I have to take part? 3

What will happen to me if I take part? 3

What will happen if I don’t want to carry on

with the study? 8

What are the possible benefits of taking part? 8

What are the possible disadvantages and risks

of taking part? 9

**Part 2**

Will I be paid to take part? 14

What to do if there are problems 14

What if new information becomes available? 15 Will my taking part in the trial be kept

confidential? 15

What will happen to the results of the study 16

Will any genetic tests be done? 16

Who is organising and funding the study? 17

Who has reviewed the study 17

How have patients been involved? 17

Further information and contact details 17

**Part 1 – Main Information Sheet**

1. **What is the purpose of the VICTOR study?**

The current standard treatment for Acute Myeloid Leukaemia (AML) has been developed and refined over the past 30 years through a series of large clinical trials (or research studies) which are planned and run on a national basis. The treatment with the best results in the previous trial is taken forward and used as the standard treatment in the next one, allowing improvements to be made step by step. Despite major progress, AML treatment is still not perfect and in particular the side effects can be extremely unpleasant or even life-threatening. The aim of this current trial is to test a new type of treatment which we hope will have less severe side effects.

The VICTOR study is for patients with AML where there is a mutation (change in the normal DNA sequence) in a gene called *NPM1*. This mutation happens by accident when cells divide and grow. It is not the kind of mutation that can be inherited or passed on. *NPM1* is one of the commonest mutations in AML and patients with this mutation usually respond well to treatment, therefore it is called ‘favourable risk’. Your doctors will have tested you for this mutation and will be able to tell you if you have it.

The VICTOR study will compare two types of treatment;

1) The standard current treatment is a combination of chemotherapy drugs and an antibody treatment (explained later) which gave the best results in previous trials.

2) The new treatment includes a drug called venetoclax, which is a drug that is very effective for treatment of other types of leukaemia and is now used routinely in the NHS for those. Venetoclax is not chemotherapy, however it makes AML cells very sensitive to small amounts of chemotherapy, meaning that much lower doses than normal have to be given, hopefully resulting in fewer side-effects. Venetoclax works by blocking a protein called BCL2, which AML cells use to resist being killed. Venetoclax given together with small doses of chemotherapy has been very effective for older patients who cannot undergo intensive chemotherapy and the results are particularly good for patients with the *NPM1* mutation.

The aim of the VICTOR study is to gather more information about how effective and safe venetoclax is compared with standard chemotherapy for patients who are healthy enough to receive treatment aimed at curing the leukaemia. If you agree to take part you will be randomly allocated to start with either standard intensive chemotherapy or venetoclax and low dose chemotherapy. With either treatment, your response will be monitored very carefully using a sensitive molecular test (performed using your blood and bone marrow sample). If the treatment is not working as well as it should, you will be able to switch treatments. For example, if you start with venetoclax treatment and tests show that the AML cells are not clearing fast enough, you will be able to switch onto to standard chemotherapy.

We will collect information about you and your disease, how it responds to treatment and any side affects you experience for 2 years after the start of your treatment.

1. **Why have I been invited to take part?**

Your doctor has invited you to consider taking part because you have been diagnosed with AML with an *NPM1* mutation (so your disease is considered ‘favourable risk’) and you are healthy enough to receive intensive treatment aimed at curing the disease. Both standard intensive chemotherapy and venetoclax with low dose chemotherapy are good treatments for AML however we don’t yet know which one is better. Approximately 186 participants from across the UK, Denmark and New Zealand will be taking part in this study. The study will take on new participants over a 2 year period. We will collect information (data) on how each patient is doing for at least 2 years. Therefore, if you agree to participate, you will be part of the study for at least 2 years.

1. **How do I enter the study?**

The first step is to decide whether you want to take part in the study. Your doctor will describe the study and talk through this information sheet with you. This information sheet is yours to take away. If you choose to enter the VICTOR study, you will be invited to sign an Informed Consent Form to show that you understand what is involved when taking part in this study.

The original signed Informed Consent Form will be placed in your hospital notes, a copy will be given to you for your records, a copy will be sent to the VICTOR Trial Office and a copy will be sent to the laboratory at Guy’s Hospital where the samples you provide as part of this study will be analysed (please see the section “What samples will be collected?” for more information).

1. **Do I have to take part?**

No, participation in this study is entirely voluntary. If you consent to participate, you are still free to withdraw from the study at any time without giving a reason. If you decide not to take part, your treatment and standard of care will not be affected in any way and your doctor will discuss your treatment options with you. For more information see the section “What will happen if I don’t want to carry on with the study?”

1. **What will happen to me if I take part?**

#### Consent and Screening

If you decide to take part in this study, we will ask you to give your written informed consent to take part and the following tests will be performed to make sure you are suitable for the study. You would have these tests as part of your routine care, whether you take part in the study or not.

- A medical history, including medicines you currently take, and those you have taken in the past
- A physical exam and vital signs assessment to measure your weight, height, temperature, pulse and performance status (assessment of how your disease effects your daily living abilities) to assess your well-being before entering the study.
- A pregnancy test (if you are a female of child bearing potential)
- Blood tests – we will collect information about the results of several different blood tests as part of this study. It is likely that you have already had most of these done. However, if any extra blood tests are needed, your nurse will let you know and will arrange this.

We will record information about the following blood tests:

- Complete blood cell count
- Assessment of kidney and liver function
- Test to look for any viruses in your blood (such as Hepatitis and HIV)

  These tests are performed to check that you are well enough to begin study treatment.
- A sample will also be sent to Guys Hospital in London for molecular testing if this has not already been done. More information about this is provided in the “What samples will be collected?” section. An additional sample will also be taken pre-treatment.
- Bone marrow aspirate and biopsy for disease assessment (a sample of the aspirate will also be sent to Guy’s Hospital in London). More information about this is provided in the “What samples will be collected?” section.
- Quality of life assessment with standardised questionnaire
- Comprehensive general health assessment (CGA), if you are 60 years of age or over. This is a series of tests taking between 15-30 minutes in total. These provide an overall picture of general health. Some of these tests are questionnaires in which you answer questions about your ability to perform day-to-day tasks and your mood. Other parts of the test involve a nurse asking you questions to assess your memory, nutrition and other health issues. Finally, you will be timed to see how long it takes you to stand up and walk.

You will need to have most of the tests described here as part of your treatment, whether or not you decide to enter this study. The extra tests that you will have as a result of entering this study are the quality of life questionnaires and the comprehensive general health assessment if you are 60 or over.

**Randomisation**

This study is a randomised controlled clinical trial. Sometimes we don’t know which way of treating patients is best. To find out, we need to compare different treatments. We put people into groups and give each group a different treatment. The results are compared to see if one is more favourable in terms of control of disease and the side effects experienced. To try to make sure the groups are the same to start with, each participant is put into a group at random by a computer. Neither you nor your doctor can choose which treatment you receive. This process is called randomisation.

You will be randomised to receive either:

- Standard intensive chemotherapy with daunorubicin, cytarabine (also called ara-c) and gemtuzumab ozogamicin (also called Mylotarg) (three different chemotherapy drugs)
- Venetoclax in combination with low-dose cytarabine (a type of chemotherapy)

#### What will happen during the study?

Whether or not you enter this study, you will be in hospital for a few weeks at the start of your treatment. If you are being treated with standard intensive chemotherapy (either in this study or not) then you will also be admitted to hospital for future treatment cycles. You will also need to attend hospital as an outpatient regularly during your treatment and afterwards.

The table below gives a description of the assessments required for this study. You would have most of these assessments as part of your routine care, whether or not you decide to enter this study. The extra tests that you will have as a result of entering this study are the quality of life questionnaires and the comprehensive general health assessment if you are 60 or over. We will try to perform these during hospital visits which were already planned as part of your treatment. You will have other visits as part of your treatment, and your doctor or specialist nurse will advise you about these.

| **Time point in the cycle** | **Tests or Procedures** | **Amount of time taken** |
| --- | --- | --- |
| Day 1 of each cycle (for the first 4 cycles) | - Full physical examination, vital signs and weight | 10 minutes |
| Weekly  (for the first 4 cycles) | - Blood tests (including blood cell count, and assessment of kidney and liver functions) | 5 minutes |
| After completion of each cycle for the first 4 cycles | - Bone marrow aspirate (including biopsy if required) and blood test to assess response to treatment. The bone marrow aspirate and blood samples will be analysed both locally and in Guy’s Hospital (more information in “What samples will be collected section”) - Quality of Life questionnaire (at the end of cycle 3 only) | 30 minutes  + 10 minutes for Quality of Life |
| Following the first 4 cycles, every month until month 12, and then every 3 months until month 24 | - Full physical examination and vital signs - Blood tests (including blood cell count) - Bone marrow aspirate (including biopsy if required) and blood test for disease monitoring (months 6, 9, 12, 15, 18, 21 and 24 only). Bone marrow aspirate and blood samples sent to Guys Hospital (more information in “What samples will be collected section”) Additional bone marrow aspirates every 4-6 weeks may be required if disease is detected after 4 cycles of treatment. - Quality of life questionnaire and performance status (months 6, 12, 18 and 24 only) - CGA if you are 60 years or over (months 12 and 24 only) | 15 minutes  + 30 minutes for bone marrow  + 10 minutes for Quality of Life  + 30 minutes for CGA |
| **End of Treatment Visit – 30 days after final treatment** | |  |
| - Blood tests (including blood cell count) and physical examination and vital signs | | 15 minutes |

#### How will the treatment be given?

Standard intensive chemotherapy arm (also called induction chemotherapy)

This treatment will be the same as that given if you choose not to take part in this study.

**Cycle 1**

You will be admitted to hospital (or will stay in hospital if you are already an inpatient) from the beginning of treatment until your blood counts recover (up to approximately 4 weeks in total). This is because the treatment will reduce your blood counts making your immune system low and leaving you vulnerable to infection. In some hospitals, you might be discharged earlier than this and followed closely in an outpatient day unit. Your doctors will explain this in more details if it applies to you. Treatment will be given intravenously (into your vein) for 10 days. The treatment will be daunorubicin, cytarabine and gemtuzumab ozogamycin (also called Mylotarg). Daunorubicin and cytarabine are both chemotherapy drugs and gemtuzumab ozogamycin is an antibody coupled to a chemotherapy drug. An antibody is a type of protein normally made by your immune system to kill germs. However, this antibody has been designed and manufactured in a laboratory to stick on to leukaemia cells and kill them. You will receive more information on the side effects of these treatments separately. You will be given daunorubicin on days 1, 3 and 5, cytarabine twice daily on days 1-10 and gemtuzumab ozogamycin on day 1 and day 4. If you have a high white blood cell count, gemtuzumab ozogamicin may be given to you on days 4 and 7.

**Cycle 2**

You will be admitted to hospital again and treatment will be given intravenously for 8 days. If you are admitted to hospital, you will usually remain an inpatient until your blood counts recover (up to approximately 4-6 weeks in total). Some hospitals may be able to discharge you earlier than this, and instead monitor you closely in an outpatient day unit. Your doctors will discuss this with you in more detail if that is an option for you. Daunorubicin will be given on days 1, 3 and 5 and cytarabine will be given twice daily on days 1-8.

**Cycles 3 and 4** (these 2 cycles are also called consolidation)

You will receive cytarabine over 5 days. You will either receive two doses per day on days 1, 3 and 5 or a single dose on days 1-5 (this depends on your age). The dose is higher than for the first two cycles. You will usually be admitted to hospital to receive this treatment, and might stay in hospital or be discharged and followed closely in an outpatient day unit, depending on your particular situation and the local arrangements. Your doctors will discuss this with you in more detail closer to the time.

Venetoclax with low dose cytarabine arm

As with the standard arm, you will usually be admitted to hospital (or will stay in hospital if you are already an inpatient) to begin your treatment although you should be able to receive cycle 2 onwards as an outpatient. Some hospitals may be able to treat you in an outpatient day unit for all cycles. Your doctors will discuss the specific details of this with you depending on your particular circumstances.

Venetoclax is given as oral capsules and your doctor will vary your dose over the first few days. Your doctor/study nurse will tell you the dose that you should be taking as this may also be affected by other medications you are taking. Venetoclax should be taken once daily, with a full glass of water within 30 minutes after breakfast or the first meal of the day. If you miss a dose, take it within 8 hours of the time you would usually take it. If the dose is missed by more than 8 hours, do not take this dose and carry on taking your capsules as normal the following day. If you are sick following taking a capsule, do not take another capsule. The next dose should be taken at the usual time the following day.

You will receive cytarabine as a subcutaneous (under the skin) injection once daily on days 1-10 of each cycle.

Venetoclax treatment will be taken for up to 2 years if your disease continues to respond. Cytarabine will be given alongside venetoclax in cycles for up to 1 year.

Both arms

To try and reduce the effect of potential side effects from these medications, your doctor will also prescribe some other medicines such as anti-sickness and antibiotics. This would be the same whether you decide to take part in this study or not. Your doctor will discuss these with you and how you should take them.

Your disease will be monitored very carefully and if it is not responding as well as it should, your treatment may be switched. This means that even if you are randomised into the venetoclax arm, you might end up having intensive chemotherapy. Similarly if you are in the intensive chemotherapy arm you might end up having treatment with venetoclax. If you do have to change treatment, the VICTOR study would still like to continue to collect information about this treatment and how you respond.

### What samples will be collected?

Bone marrow samples

Having bone marrow tests is an essential part of monitoring your response to treatment, and you will need to have these whether you enter this study or not.

There are two types of bone marrow tests: bone marrow aspirate and bone marrow biopsy.

- A bone marrow aspirate is the removal of a small sample of liquid bone marrow.
- A bone marrow biopsy is the removal of a small solid piece of bone marrow which is about the thickness of a pencil lead and about 1-2cm (half an inch to an inch) long.

Whether or not you enter this study, you will need to have both types of bone marrow sample taken before starting treatment. After each cycle of treatment, you will usually only have a bone marrow aspirate however your doctors might need to do a biopsy as well in certain situations.

Whether or not you enter this study, you will also need to have follow-up bone marrow aspirates done every three months until two years after your treatment has finished. If after 4 cycles of treatment, your bone marrow aspirates show that your disease is still detectable, you may need to have further bone marrow aspirates every 4-6 weeks until your disease is no longer detectable. This allows very close monitoring of your disease and allows further treatment to be planned if the leukaemia starts to come back (this is called relapse).

All your bone marrow samples will be tested using a very accurate method called a minimal residual disease (MRD) test. This test can usually detect one leukaemia cell mixed in with a million normal cells. If you don’t enter this study your doctors will usually still recommend MRD testing for at least two years after treatment as standard, as this allows relapses to be detected at a very early stage.

For this study, bone marrow aspirate samples will be sent to Guys Hospital, where the MRD analysis will take place. About 5mls (approximately 1 teaspoon) of bone marrow will be taken for this. The results will be provided to your doctors within two weeks and will be used to help plan your treatment. As well as this, a small drop of bone marrow will also be spread onto a glass slide and this will be looked at locally by your own study doctor or somebody in their team.

Blood samples

Blood samples will be taken at the time points specified in the treatment schedule above. These will be taken whether you enter the study or not to monitor your disease and response to treatment, to help inform treatment options for you. We will collect an additional 20mls of blood (approximately 4 teaspoons) at the following time points to send to Guys Hospital; screening (to test for eligibility for the study), baseline (at any time pre-treatment), the end of each cycle of treatment for the first 4 cycles, cycle 6, then every 3 cycles for 2 years from the time you started treatment. These samples will be taken at the same time as your routine blood test to avoid the discomfort of multiple blood draws.

**What will happen to the samples at the end of the study?**

We will need to keep samples of blood and bone marrow until the end of the study for all patients in case there are any discrepancies in the results and re-testing is needed.

If you agree, we would like to keep samples of your blood and bone marrow for a sub-study. This sub-study is completely optional, and you can still take part in the VICTOR study without participating in this sub-study. During the sub-study, researchers at Guy’s hospital would like to analyse the blood and bone marrow from patients who enter a deep remission without having intensive chemotherapy. This data will be used to better understand and develop patient-specific treatment for AML.

Lastly, if you agree, we would like to keep samples of your blood and bone marrow after the VICTOR study has finished to allow further research on AML for example to test new treatments or to identify new markers that predict response. Donation of your samples for existing or future research is optional, and you can still enter the study without agreeing to this.

If you agree to donate your samples, after the study has finished any remaining material will be stored at Guy’s Hospital in a Tissue Bank. These samples may be analysed in an existing study or in the future as part of a new research study. Any research carried out on the samples will need to have ethical approval. Any samples stored will be anonymous and will not be able to be linked with you personally.

If you do not agree to donate your samples, these will be safely destroyed at the end of the study.

1. **What will happen if I don’t want to carry on with the study?**

You are free to withdraw from this study at any time, you do not have to give a reason and your future care will not be affected. If you decide to withdraw from the study treatment your doctor may ask you to return to hospital for follow up assessments for safety reasons. Your doctor will then discuss your further treatment options with you.

If you choose to withdraw from the study, we would still retain and analyse any information and samples we have collected up until withdrawal. If you decide to withdraw from the treatment only, you can decide whether to allow your hospital to continue to send information about your progress to the Trial Office. You will also be asked to return any unused medication, if applicable.

1. **What are the possible benefits of taking part?**

There is no guaranteed benefit to taking part in this study because we do not yet know which of the two treatments is better and it is possible that the new treatment is not as good as the standard treatment. Equally, it is possible that the standard treatment is not as good which is why this study is being done. The careful monitoring you will receive if you take part in this study is a safeguard against this risk. As the new treatment is likely to be less toxic, it is possible that patients receiving this treatment may experience fewer side effects. The information gained from this study will help improve treatment for other people with AML in the future.

1. **What are the possible disadvantages and risks of taking part?**

**What are the risks of the bone marrow aspirates and biopsies?**

You will need to have bone marrow aspirate and biopsy procedures performed whether or not you enter this study. Both of these processes may be painful but you will be given a local anaesthetic to numb the area before the tests are performed. However, it may be a little uncomfortable afterwards. The bone marrow aspirate takes approximately 5-10 minutes and if a biopsy is also taken this will take an extra few minutes. You will normally have to lie down afterwards for half an hour. Please tell your doctor if you have any pain and you will be given painkillers.

The area will be covered with either a sticky plaster or a gauze pad. There may be a small amount of bleeding which is perfectly normal, but the doctor or nurse will make sure this has stopped before you go home.

If the site does start to bleed again, press on the area with a clean cloth or handkerchief. By pressing on the area, this will help your blood to clot and the bleeding to stop. The possible side effects associated with a bone marrow biopsy include pain, bleeding, bruising and infection, as well as a reaction to the numbing agent.

**What are the side effects of treatment?**

It is very likely that you will experience side effects from your treatment whether you take part in this study or not. In particular, both treatments kill all healthy cells within the bone marrow as well as the leukaemia cells. This means that you will not be able to produce normal numbers of red blood cells and platelets and you are likely to need transfusions of these from blood donors and may be at risk of bleeding especially if platelet transfusions are missed or delayed. More importantly the white blood cell count will fall, usually to zero, meaning that you will be at high risk of serious infections. Your doctor will prescribe antibiotics to prevent these and will instruct you about the steps to be taken if you develop a high temperature. If you do develop a temperature this can quickly turn into a life-threatening infection without prompt treatment so it is important that you receive medical attention and antibiotics straight away. Infection is the most common cause of treatment-related death for patients with AML.

Although low blood counts and infection are the most serious side effects of leukaemia treatment there are a number of other possible side effects listed below. Your doctor will explain these in more detail before you have any treatment.

You should tell your study doctor or medical team about any side effects that you have, even if you do not think they are connected to the drugs. Your doctor may be able to give you medications to help treat the side effects and prevent them from becoming worse. All side effects will be monitored closely to minimise any risks to you. Your study doctor may also choose to stop or delay treatment for a short time or reduce the dose to allow you to recover from any side effects.

**Side effects associated with venetoclax**

Side effects that have been seen in at least 1 in 10 participants (very common) include

- loose or watery stools (diarrhoea)
- constipation
- Nausea
- Vomiting
- Tiredness (fatigue)
- Infections
- Sepsis
- Decreased appetite
- Low potassium levels in your blood (can lead to muscle cramps or weakness and abnormal heart rhythms
- Dizziness and headaches
- Low blood pressure
- Laboured breathing (dyspnoea)
- Joint pain
- Weight loss
- Abdominal pain
- Inflamed and sore mouth
- Increased bilirubin levels due to changes in your liver function

Side effects that have been seen in at least 1 in 100 participants (common) include

- Unusual levels of chemicals in the blood caused by the fast breakdown of cancer cells, which may lead to changes in kidney function, abnormal heartbeat, or seizures (Tumour Lysis Syndrome (TLS)).Gall stones and inflammation of the gall bladder

**Side effects associated with cytarabine**

Side effects that have been seen in at least 1 in 10 participants (very common) include

- Inflammation or appearance of sores in the mouth, lips, or on the anus (back passage)
- Nausea or vomiting
- Diarrhoea
- Abdominal pain
- Liver damage
- Hair loss is common and may be quite severe. Hair normally re-grows when your treatment course ends.
- Skin rash
- Cytarabine syndrome; sometimes the following side effects can happen together 6 to 12 hours after receiving Cytarabine: feeling generally unwell with a high temperature, bone pain, muscle and sometimes chest pain, blistery rash and/or sore eyes. This is called "Cytarabine Syndrome" and can be treated.
- Feeling hot and feverish
- Abnormal bone marrow or blood test results (these will be monitored throughout treatment)

Side effects that have been seen in at least 1 in 100 participants (common) include

- Ulceration on your skin

**Side effects associated with daunorubicin**

The frequency of the following events is not known. Some events may be very common, some may be rare. Your doctor can provide further information on how likely you are to experience them.

- Unusual levels of chemicals in the blood caused by the fast breakdown of cancer cells, which may lead to changes in kidney function, abnormal heartbeat, or seizures (Tumour Lysis Syndrome). Patients with very high white blood cell counts are at higher risk of tumour lysis syndrome.
- Secondary leukaemia has been reported in association with daunorubicin when used in combination with other anti-cancer drugs.
- Heart disease (cardiomyopathy). Daunorubicin may cause permanent damage to the heart muscle which may in some cases cause symptoms which might be severe. To minimise this risk, you will usually have an echocardiogram performed before any treatment to make sure there are no pre-existing problems. If there are, your doctor may decide daunorubicin treatment is not suitable for you. If problems develop during or after treatment you may need to have a second echocardiogram and advice may be sought from a cardiologist (heart specialist) which in some cases may include stopping treatment with daunorubicin.
- Inflammation of the mucous membranes such as the mouth, nose, eyelids, windpipe/lungs and stomach/intestines (mucositis/stomatitis); symptoms can include pain, burning sensation, redness, ulcerations, bleeding and infections.
- Diarrhoea
- Nausea/vomiting
- Abdominal pain
- Inflammation of the lining of the colon (colitis)
- Hair loss is common and may be quite severe. Hair normally re-grows when your treatment course ends.
- Skin problems such as redness, rash, itching, hives and pigment changes
- Kidney damage
- Red colour of urine for 1 to 2 days after administration
- Reduced fertility in both men and women
- Premature menopause in women
- Drug may leak into the surrounding tissue causing immediate local pain/burning sensation, severe skin infection, painful ulceration, inflammation and tissue death. For this reason the drug will normally be given to you through a special line (e.g. PICC line or Hickman line).
- Liver damage

**Side effects associated with gemtuzumab ozogamicin**

Side effects that have been seen in at least 1 in 10 participants (very common) include

- Impaired liver function
- Unusual levels of chemicals in the blood caused by the fast breakdown of cancer cells, which may lead to changes in kidney function, abnormal heartbeat, or seizures (Tumour Lysis Syndrome). Patients with very high white blood cell counts are at higher risk of tumour lysis syndrome.
- High blood sugar
- Increased time it takes the blood to clot (increased prothrombin time)

Side effects that have been seen in at least 1 in 100 participants (common) include

- Venoocclusive liver disease; a condition where small veins in the liver are obstructed. Symptoms can include weight gain, abdominal pain, liver swelling and yellow discoloration of the skin. Please tell your doctor immediately if you develop any of these symptoms. In some cases veno-occlusive disease may be fatal. Your doctors will only give you gemtuzumab ozogamicin if tests on your liver are satisfactory, you will be carefully monitored for this condition by blood tests and regular physical examination and treatment will be started promptly if it is suspected.

**All treatments**

Allergic reactions

Sometimes people have allergic reactions to drugs. Serious allergic reactions can be life-threatening. If you have an allergic reaction to one of these drugs, you might develop a rash, difficulty breathing, wheezing when you breathe, sudden low blood pressure with light-headedness, swelling around the mouth, throat or eyes, a racing heartbeat, and/or sweating. Before starting the study drug, you must tell your Study Doctor about any drug allergies. You should tell the Study Doctor right away if you have any allergy symptoms listed above.

Fatigue

If you are affected by fatigue caution must be exercised when driving or using heavy machinery. Do not carry out these activities if you feel it is not safe to do so.

**Harm to the unborn child**

Information for Women

All of the drugs in this study may cause harm to an unborn child if administered during pregnancy. There is little or no information of the effects on the child when breast feeding during treatment with any of the drugs in this study.

You cannot take part in this study if you are pregnant, breast-feeding, planning to become pregnant or to do an egg donation while receiving study medication until 6 months after the last study drug administration, or 30 days following venetoclax discontinuation if you are receiving venetoclax alone (whichever is later). If you are a female who can become pregnant, you will be asked to take a pregnancy test prior to starting study drug treatment.

If you decide to take part in this study, you must agree to use two forms of effective contraception without interruption, including one barrier method (see box below).

If you become pregnant while receiving study medication or within 6 months after the last study drug administration (or within 30 days if you are taking venetoclax alone), you must tell your doctor right away and any study medication you are taking will be discontinued. Your doctor will explain how to safeguard your health and the health of your baby. If you agree, we will collect Information related to the progress of your pregnancy and its outcome that is relevant to the study. This may include information related to your health, the date of conception, the course and outcome of your pregnancy and any medical treatments that you receive.

Information for Men

If you were to father a child, the treatments you will receive as part of this study may be harmful to the unborn child.

If your partner might become pregnant you/your partner must agree to use two effective forms of contraception, including one barrier method (see box below) during the trial treatment and for 6 months after the last study drug administration, or 30 days following venetoclax discontinuation if you are receiving venetoclax alone (whichever is later). Your doctor will talk to you about potential sperm donation before you start treatment and you should not be involved in sperm donation during this time. If your partner becomes pregnant during the course of the study, we would ask you to tell your study doctor immediately and your doctor will ask you and your partner for permission to collect information about the pregnancy and the child. Your doctor will explain to you and to your partner, how to safeguard your partner’s health and the health of the baby.

The acceptable methods of effective contraception are: combined hormonal contraception or progestogen-only hormonal contraception associated with inhibition of ovulation, intrauterine device (IUD), intrauterine hormone-releasing system (IUS), bilateral tubal occlusion (a surgical procedure that involves blocking the fallopian tubes), a vasectomised partner, or sexual abstinence.

Barrier methods include: condom, diaphragm, cervical cap and contraceptive sponges with spermicide.

**Please speak to your study doctor who will be able to provide appropriate contraception advice.**

**What food/medications (additional medication) should I avoid?**

**It is very important that you tell the study doctor about all medications, supplements, or herbal medicine that you are taking now and during the study.** Even herbal medicines and other alternative treatments can interact with chemotherapy, venetoclax and the medicines you will be given to prevent infections, and these interactions could be dangerous.

The following information in this section is only relevant to patients taking venetoclax.

Some juices or foods, as well as some medications, may interfere with the way your body processes venetoclax. This interference could cause the amount of the drug in your body to be higher or lower than expected.

The following must be avoided:

| **Foods to avoid** |
| --- |
| Grapefruit and grapefruit juice, Seville oranges (including marmalade and juice), pomelos, star fruit. |
| **Herbal supplements to avoid** |
| St. John’s Wort and curcumin |
| **Be sure to tell your study staff immediately about any side effects and new medications to avoid possible harm.** |

# Part 2 – Additional Information

1. **Will I be paid to take part?**

You will not receive any money for taking part in this study and unfortunately travel expenses cannot be reimbursed by the study organisers. However, some hospitals may offer travel expenses if you take part in the study and other transport services **may** be available. Please discuss access to these with your medical team.

1. **What to do if there are problems**

The main concern of everyone involved with this study is that your treatment is as safe, effective and tolerable as possible. If you have a concern about any aspect of this study, you should ask to speak to your doctor or study nurse who will do their best to answer your questions.

**Complaints**

If you remain unhappy and wish to complain formally, you can do this through the NHS Complaints Procedure. Details can be obtained from your hospital. Additionally, the contact information for your local Patient Advice and Liaison Service (PALS) or equivalent is at the end of this information sheet.

**If you are harmed**

If you are harmed by taking part in this study due to someone’s negligence, then you may have grounds for a legal action for compensation but you may have to pay your legal costs. NHS Trusts have a duty of care to participants whether or not the participant is taking part in a clinical trial and normal National Health Service complaints mechanisms will still be available to you. If you have private medical insurance, you should tell your insurer that you are taking part in research. They will let you know if it affects your policy.

1. **What if relevant new information becomes available?**

If we get new information about the treatment being studied, your doctor will tell you and discuss whether you should continue in the study. If you decide not to carry on, your doctor will make arrangements for your care to continue. If you decide to continue in the study your doctor may ask you to sign an updated Informed Consent Form. If new information becomes available your doctor might consider that you should withdraw from the study. Your doctor will explain the reasons and arrange for your care to continue. If the study is stopped for any other reason, we will tell you and arrange your continuing care so you receive the best care available.

1. **Will my taking part in the study be kept confidential?**

All information collected about you for this study will be subject to the General Data Protection Regulation and Data Protection Act 2018 and will be kept strictly confidential.

All information collected by the Sponsor will be securely stored at the Trial Office at the Cancer Research UK Clinical Trials Unit, University of Birmingham on paper and electronically and will only be accessible by authorised personnel associated with the trial. The only people in the University of Birmingham who will have access to information that identifies you will be people who manage the study or audit the data collection process. When you are entered into the study we will collect your date of birth and initials. You will be given a unique study number and in routine communication between your hospital and the Trial Laboratory and Trial Office, you will only be identified by this study number. A copy of your signed consent form will be posted to the Trial Office to ensure that the correct consenting procedure has been carried out. A copy of your signed consent form will also be sent to the laboratory at Guys Hospital so that they have confirmation that you have consented to your samples being analysed. This will have your name and signature on it.

The NHS will use your name and contact details to contact you about the research study, and make sure that relevant information about the study is recorded for your care, and to oversee the quality of the trial.

If you live in England, you may have been asked by your doctor to decide if you would like to participate in the National Genome Research Library. This is a secure national database of de-identified genomic and health data managed by Genomics England. If you have agreed to participate in the National Genome Research Library, with your consent, approved researchers will be able to see that you are a patient on the VICTOR study. **The data in the National Genome Research Library is updated throughout your lifetime for as long as you have given your permission**. The National Genome Research Library uses patients’ NHS number and date of birth. On the VICTOR Informed Consent Form we will ask you specifically for consent to collect your NHS number and date of birth to link your medical data collected as part of the study with the genome sequencing data and de-identified clinical data stored within the National Genome Research Library. If you do not wish to provide this, it will not affect your participation in VICTOR or the care you receive.

Samples taken for research purposes and sent to Guys Hospital will be identified by your unique study number, date of birth and initials. This information is the minimum needed to make sure that your samples can be identified as yours.

In addition anonymised data (i.e. with patients initials removed) from the trial may be provided to other 3rd parties (e.g. pharmaceutical companies or other academic institutions) for research, safety monitoring or licensing purposes. This includes sending data to the manufacturers of the drugs (AbbVie) for safety monitoring purposes. They have the same duty of confidentiality to you as other personnel. These organisations could be within Europe, or outside Europe where the data protection laws may be different. Data sent abroad will not allow you as an individual to be identified.

By taking part in the study, you will be agreeing to allow research staff from the Trial Office at the University of Birmingham to look at the study records, including your medical records that are relevant to this study. It may be necessary to allow authorised personnel from government regulatory agencies (e.g. Medicines and Healthcare products Regulatory Agency (MHRA)), the Sponsor and/or NHS bodies to have access to your medical and research records. This is to ensure that the study is being conducted to the highest possible standards.

From time to time we may be asked to share the trial information (data) we have collected with researchers running other studies in this organisation and in other organisations so that they can perform analysis on the data to answer other important questions about AML. These organisations may be universities, NHS organisations or companies involved in health research and may be in this country or abroad. Any such request is carefully considered by the study researchers and will only be granted if the necessary procedures and approvals are in place. This information will not identify you and will not be combined with other information in a way that could identify you. The information will only be used for the purpose of health research, and cannot be used to contact you or to affect your care. It will not be used to make decisions about future services available to you, such as insurance. Under no circumstances will you be identified in any way in any report, presentation or publication arising from this or any other study.

You can withdraw your consent to our processing of your data at any time. Under the provisions of the General Data Protection Regulation (GDPR) 2018, you have the right to know what information the Trial Office has recorded about you. If you wish to view this information, or find more about how we use this information, please contact Legal Services at the address below or email [dataprotection@contacts.bham.ac.uk](mailto:dataprotection@contacts.bham.ac.uk).

Legal Services
University of Birmingham
Edgbaston
Birmingham, B15 2TT

**Involvement of the General Practitioner (GP) /Family Practitioner**

It is important that your GP is kept up to date with any treatment you are receiving. Your GP will be informed that you are taking part in this research study and they will be sent a copy of this information sheet.

1. **What will happen to the results of the study?**

When the study is complete the results will be published in a medical journal but no individual patients will be identified. If you would like to have a copy of the published results, please ask your study doctor or nurse.

1. **Will any genetic tests be done?**

Yes, please refer to the “Why have I been invited to take part?” section. In addition to genetic testing at screening, researchers will also monitor the level of tumour cells in your bone marrow to see if you are responding to treatment. Results and data from analysing the samples as part of the study will be returned to your local doctor and used to guide your medical care. These tests only look for genetic changes which occurred during your life-time. They do not look for inherited genetic problems and so these results will not have consequences for your family members.

1. **Who is organising and funding the study?**

This research is being funded by Cancer Research UK with contributions from AbbVie and Cure Leukaemia for Research Nurse support. AbbVie are providing free venetoclax for the study and they have provided an educational grant to cover the cost of the laboratory work that will be carried out. The study is being run by the TAP hub at the Cancer Research UK Clinical Trials Unit at the University of Birmingham.

1. **Who has reviewed the study?**

This research study has been reviewed by Cancer Research UK, the Cancer Research UK Clinical Trials Unit and also by an independent Research Ethics Committee. Research Ethics Committees review all research to protect the safety, rights, wellbeing, and dignity of participants. This study was reviewed and received favourable opinion by the London Bridge Research Ethics Committee. It has been reviewed and received a Clinical Trial Authorisation by the UK Competent Authority (Medicines and Healthcare products Regulatory Agency - MHRA) It has also been reviewed by the national Health Research Authority.

1. **How have patients been involved in this study?**

The Trial Management Group for this study has two patient representatives who have lived experience of AML and its treatment. They have provided advice on the trial design and reviewed this information sheet and helped us to develop it so that it is clear and correct. They will also be involved in the ongoing management of this study, in order to make sure that the interests of patients are maintained.

1. **Further information and contact details**

If you have any questions or concerns about your disease or this research study, please discuss them with your doctor. You may also find it helpful to contact the following organisations:

*<< insert name and contact telephone number of Principal Investigator >>*

*<< insert name and contact telephone number of Research Nurse >>*

*<< insert 24 hour emergency contact details >>*

*<<Delete as appropriate for your site>>*

You may also find it helpful to contact the following organisations:

**England**

Your local Patient Advice and Liaison Service (PALs) or equivalent who provide advice and support to patients, their families and their carers, website: <http://www.nhs.uk/chq/Pages/1082.aspx?CategoryID=68&SubCategoryID=153>

Or local PALS details where available:

*<< insert address and contact telephone number of local service >>*

**Northern Ireland**

In Northern Ireland the Patient Client Council (PCC) can provide assistance and support at any stage of the health and social care services complaints procedure. The PCC is an independent body who represent the views of the public in all areas of health and social care. They can also assist you to make a complaint. This is a confidential and free service.

<http://www.patientclientcouncil.hscni.net/>

Telephone: 0800 917 0222

Email: [info.pcc@hscni.net](mailto:info.pcc@hscni.net)

**Scotland**

The Patient Advice and Support Service is an independent service which provides free, accessible and confidential information, advice and support to patients, their carers, and families about NHS healthcare in Scotland.

<http://www.patientadvicescotland.org.uk/>

**Wales**

Community Health Councils (CHCs) are independent bodies, set up by law, who listen to what individuals and the community have to say about the health services with regard to quality, quantity, access to and appropriateness of the services provided for them. CHCs can also help, advise and support people who wish to make complaints about NHS services and similar matters. This advice is completely free, independent and confidential.

<http://www.wales.nhs.uk/sitesplus/899/home>

**Sources of information**

**CancerHelp:** an information service about cancer from Cancer Research UK, Freephone 0808 800 40 40, [www.cancerhelp.org.uk](http://www.cancerhelp.org.uk)

**Macmillan Cancer Support:** Freephone 0808 800 0000, [www.macmillan.org.uk](http://www.macmillan.org.uk)

**CRUK Clinical Trial Database:** <https://www.cancerresearchuk.org/about-cancer/find-a-clinical-trial>

**Emergencies**

**If a medical emergency, related to your treatment for this study occurs while you are at home, you should initially try to contact the haematology unit where you received your treatment (see contact details below). If this is not possible you should go to the accident and emergency (A&E) department at your local hospital. If you are unable to get to the hospital you should contact your GP who will already have been informed of your participation in the study.**

Please take as much time as you need to make a decision and then let your doctor know what you have decided so that your treatment can be arranged.

**Thank you for taking time to read this leaflet and considering taking part in this study. You may use this information sheet to make notes or write down any questions you may have.**

# ****Blood Sample Analysis at Guy’s Hospital Patient Information Sheet****

#

*Print on hospital headed paper*

**What am I being asked to do?**

You have been advised by your doctor to have a sample of your blood taken for routine diagnostic reasons. Researchers would like this sample to be analysed at Guy’s Hospital in London rather than your routine laboratory to see if you would be eligible to take part in a clinical study called VICTOR which is looking at a new treatment for patients with a specific type of Acute Myeloid Leukaemia that affects around one third of patients.

**Do I have to provide a blood sample?**

No. If having read this information sheet you decide not to allow a blood sample to be analysed at Guy’s Hospital, your standard of care will remain the same.

**What will happen if I agree to provide a blood sample?**

You will be asked to sign a consent form. You will be given a copy to keep along with this Patient Information Sheet. When you have a routine blood sample taken your doctor will take extra blood (20mls of blood (approximately 4 teaspoons)), specifically for analysis at Guy’s Hospital. If you are having a bone marrow biopsy performed as part of your care, this may also be sent to the laboratory. This is so that if you end up entering the VICTOR trial, you do not have to have a repeat bone marrow biopsy just for the trial entry requirements.

**Where will my sample be kept?**

Your blood sample (and bone marrow sample if applicable) will be stored at Guy’s Hospital if you are suitable for and subsequently consent to go into the VICTOR study. If you do not go into the VICTOR study, your sample may still be useful for molecular testing later in your treatment.

**What will happen to any left-over samples?**

If you agree, we would like to keep any left-over samples to allow further research on AML for example to test new treatments or to identify new markers that predict response. Donation of your samples for existing or future research is optional, and you can still enter the study without agreeing to this. If you agree to donate your samples any remaining material will be stored at Guy’s Hospital in a Tissue Bank. These samples may be analysed in an existing study or in the future as part of a new research study. Any research carried out on the samples will need to have ethical approval. Any samples stored will be anonymous and will not be able to be linked with you personally.

**What are the benefits?**

Analysis of your blood sample will allow researchers to identify which genes have been affected as part of your disease. This information will be sent back to your local doctor within 24 hours of the lab receiving the sample and will allow your doctor to make decisions about which treatment is best for you. This may include the option to enter the VICTOR study and further information will be provided to you if this is a possibility.

**What are the risks?**

The physical risks are the same as having a routine blood test. Having blood taken may cause some discomfort, bleeding or bruising where the needle enters the body and, in rare cases, light-headedness and fainting.

**What about confidentiality?**

Hospital and laboratory staff are the only people who will know your identity. A copy of your signed consent form will be sent to the laboratory at Guys Hospital. This is so the laboratory has confirmation that you have consented to have your samples analysed and to enable your sample to be linked to you to provide the results back to your doctor. Your hospital may be asked to provide information on your disease along with your samples sent to the laboratory. This information will not identify you and will not be combined with other information in a way that could identify you. The information will only be used for the purpose of health research, and cannot be used to contact you or to affect your care. It will not be used to make decisions about future services available to you, such as insurance. Under no circumstances will you be identified in any way in any report, presentation or publication arising from this or any other study.

**What happens if I change my mind?**

Once you have provided a sample you can change your mind at any time before entering the VICTOR study by contacting your hospital. As this analysis happens quickly, it may be too late to stop the sample being looked at but any remaining sample or information about the results held at Guy’s Hospital can be destroyed.

Your doctor or nurse will be happy to answer any questions you have about providing a blood sample.

**Contact Details**

Name: _______________________________________________________

Telephone number: _______________________________________________________

# Patients to be given a copy of this Patient Information Sheet

# and a signed copy of the Informed Consent Form to keep
