# Supplementary material for: Venetoclax combined with low dose cytarabine compared to standard of care intensive chemotherapy for the treatment of favourable risk adult acute myeloid leukaemia (VICTOR): Study protocol for an international, open-label, multicentre, molecularly-guided randomised, phase II trial
Source: BMC Cancer. 2022 Nov 14;22:1174. doi: 10.1186/s12885-022-10221-2 (PMC9664612; doi:10.1186/s12885-022-10221-2)
Supplement: Supplementary file 1 — Additional file 1: Supplementary Appendix 1. SPIRIT checklist for the VICTOR protocol A completed Standard Protocol Items: Recommendations for Intervention Trials (SPIRIT) checklist for the VICTOR protocol. Supplementary Appendix 2. WHO trial registration data set for the VICTOR trial The World Health Organization (WHO) trial registration data set for the VICTOR trial. Supplementary Appendix 3. VICTOR informed consent forms Exemplar informed consent and blood sample analysis consent form for the VICTOR trial. Supplementary Appendix 4. VICTOR patient information sheets Exemplar trial and blood sample analysis patient information sheets for VICTOR. Supplementary Appendix 5. VICTOR schedule of events Patient schedule of events for the VICTOR trial. Supplementary Appendix 6. Adverse event definitions Definitions of adverse events used for the VICTOR trial. [file 12885_2022_10221_MOESM1_ESM.zip › VICTORprotocol_Appendix5 v1.0R2.docx]

# Supplementary Appendix 5: VICTOR schedule of events

**.**

|  | **Screening** (28 days) | **Cycle 1** | | | | | **Cycles 2-4** | | | | |  | **Months 5-12^1^** | | | | | **Months 13-24** | | | | **28 days after last treatment** |
| --- | --- | --- | --- | --- | --- | --- | --- | --- | --- | --- | --- | --- | --- | --- | --- | --- | --- | --- | --- | --- | --- | --- |
|  |  | **Day 1** | **Day 8** | **Day 15** | **Day 22** | **Day 29-42** | **Day 1** | **Day 8** | **Day 15** | **Day 22** | **Day 29-42** | **Month 5** | **Month 6** | **Month 7 and 8** | **Month 9** | **Month 10 and 11** | **Month 12** | **Month 15** | **Month 18** | **Month 21** | **Month 24** |  |
| Informed consent | X |  |  |  |  |  |  |  |  |  |  |  |  |  |  |  |  |  |  |  |  |  |
| Medical history | X |  |  |  |  |  |  |  |  |  |  |  |  |  |  |  |  |  |  |  |  |  |
| Pregnancy test (if applicable) ^2^ | X |  |  |  |  |  | X |  |  |  |  | X | X | X | X | X | X |  |  |  |  |  |
| Physical exam (Height (baseline only), Temp, BP, Pulse) | X | X |  |  |  |  | X |  |  |  |  | X | X | X | X | X | X | X | X | X | X | X |
| ECOG performance status | X | X |  |  |  |  |  |  |  |  | Cycle 3 only |  | X |  |  |  | X |  | X |  | X |  |
| Weight and BSA^3^ |  | X |  |  |  |  | X |  |  |  |  | X | X | X | X | X | X |  | X |  | X |  |
| Bone Marrow aspirate (+/- trephine) and blood sample | X^4^ |  |  |  |  | X^5^ |  |  |  |  | X^5,6^ |  | X^5^ |  | X^5^ |  | X^5^ | X^5^ | X^5^ | X^5^ | X^5^ |  |
| Response assessment |  |  |  |  |  | X |  |  |  |  | X |  | X |  | X |  | X | X | X | X | X |  |
| Haematology ^6^ | X | X | X | X | X | X | X | X | X | X | X | X | X | X | X | X | X | X | X | X | X | X |
| Biochemistry ^7^ | X | X | X | X | X | X | X | X | X | X | X |  |  |  |  |  |  |  |  |  |  |  |
| Virology (HIV, HBV, HCV) | X |  |  |  |  |  |  |  |  |  |  |  |  |  |  |  |  |  |  |  |  |  |
| CGA^8^ for patients ≥60 | X |  |  |  |  |  |  |  |  |  |  |  |  |  |  |  | X |  |  |  | X |  |
| QoL | X |  |  |  |  |  |  |  |  |  | Cycle 3 only |  | X |  |  |  | X |  | X |  | X |  |
| Concomitant medications |  |  | << Ongoing assessment until treatment discontinuation >> | | | | | | | | | | | | | | | | | | |  |
| Adverse events^9^ |  |  | << Ongoing assessment>> | | | | | | | | | | | | | | | | | | | |
| Supportive Care Requirements^10^ |  |  | << Ongoing assessment until treatment discontinuation >> | | | | | | | | | | | | | | | | | | |  |

|  | **Cycle 1** | | | | | | | | | | **Cycle 2** | | | | | | | | **Cycle 3 and 4** | | | | |
| --- | --- | --- | --- | --- | --- | --- | --- | --- | --- | --- | --- | --- | --- | --- | --- | --- | --- | --- | --- | --- | --- | --- | --- |
|  | **Day 1** | **Day 2** | **Day 3** | **Day 4** | **Day 5** | **Day 6** | **Day 7** | **Day 8** | **Day 9** | **Day 10** | **Day 1** | **Day 2** | **Day 3** | **Day 4** | **Day 5** | **Day 6** | **Day 7** | **Day 8** | **Day 1** | **Day 2** | **Day 3** | **Day 4** | **Day 5** |
| **Treatment arm 1 –** DAGO^11^ | | | | | | | | | | | | | | | | | | | | | | | |
| Cytarabine | X | X | X | X | X | X | X | X | X | X | X | X | X | X | X | X | X | X | X | (X)^14^ | X | (X)^14^ | X |
| Daunorubicin | X |  | X |  | X |  |  |  |  |  | X |  | X |  | X |  |  |  |  |  |  |  |  |
| Gemtuzumab ozogomicin ^12^ | X |  |  | X |  |  | X^12^ |  |  |  |  |  |  |  |  |  |  |  |  |  |  |  |  |

|  | | **Cycle 1** | | | | | | **Cycles 2-4** | | | | | **Months 5-12^1^** | | | | | | **Months 13-24** | | | |
| --- | --- | --- | --- | --- | --- | --- | --- | --- | --- | --- | --- | --- | --- | --- | --- | --- | --- | --- | --- | --- | --- | --- |
|  | | **Day 1** | **Day 8** | **Day 15** | | **Day 22** | **Day 28** | **Day 1** | **Day 8** | **Day 15** | **Day 22** | **Day 28** | **Month 5** | **Month 6** | **Month 7 and 8** | **Month 9** | **Month 10 and 11** | **Month 12** | **Month 15** | **Month 18** | **Month 21** | **Month 24** |
| **Treatment arm 2 –** VEN+LDAC^11^ | | | | | | | | | | | | | | | | | | | | | | |
| Venetoclax^13^ | X | | X | X | x | | x | X | X | X | x | x | X | X | X | X | X | X | X | X | X | X |
| Cytarabine | D1-10 | | | |  | |  | D1-10 | | |  |  | D1-10 of each cycle | | | | | |  |  |  |  |
| Aciclovir, voriconazole (or posaconazole) | X | | X | X | X | | X | Prophylaxis may continue at the discretion of the Investigator (see section 7 for details) | | | | | | | | | | | Prophylaxis is not recommended during venetoclax maintenance | | | |

**Key**

- - - 1. Patients to be seen on day 1 of each cycle during months 5-12. Additional clinic visits at the discretion of the treating Investigator in line with local treatment protocols.
      2. Where applicable (in females of child-bearing potential), every effort should be made to perform the screening pregnancy test within 14 days of the first dose of trial treatment. If the screening pregnancy test exceeds 14 days prior to the first dose of treatment, the test must be re-taken prior to the patient receiving treatment. Pregnancy test must be re-taken prior to each treatment cycle in females of child-bearing potential.
      3. Body Surface Area (BSA) to be calculated according to local practice. Weight to be taken on day 1 of cycles 2-4 for all patients and at the start of each subsequent cycle up to cycle 12 for venetoclax and low-dose cytarabine (VEN+LDAC) patients. BSA to be recalculated if necessary, depending on weight changes as per local practice. Weight also to be taken at months 12 and 24 for CGA and body mass index (BMI) assessment.
      4. Baseline bone marrow (BM) assessment includes aspirate for local cytogenetics, flow cytometry and morphology. Trephine performed for local histopathology. A baseline BM aspirate sample should be sent to Guy’s Hospital. A blood sample should be to be sent to Guys Hospital for genotype assessment for eligibility prior to enrolment. If the patient is eligible and goes into the trial, a further baseline blood sample should be sent with the baseline bone marrow aspirate prior to starting treatment.
      5. Perform upon count recovery (where applicable). If counts do not recover, perform on day 42. BM includes aspirate sample for central molecular monitoring and an aspirate slide for local morphology. Repeat if sample inadequate or concerning result. Trephine only performed for evidence of morphological leukaemia free state in cases with delayed count recovery. Paired blood and bone marrow aspirate samples to be sent to Guy’s Hospital for minimal residual disease (MRD) monitoring. Disease status must be confirmed before continuing treatment.
      6. If the patient’s BM aspirate is MRD positive after cycle 4, but the reduction is more than 4 log_10_ the patient should have BM aspirates checked every 4-6 weeks until MRD negativity is achieved. If the post cycle 4 BM aspirate is MRD negative, the frequency of BM aspirates may be reduced to every 3 months.
      7. Haematology to include: absolute neutrophil count, haemoglobin, lymphocytes, platelets, white cell count. Can be performed +/- 3 days.
      8. Biochemistry to include: Albumin, alkaline phosphatase, alkaline transferase, aspartate aminotransferase, bicarbonate, bilirubin, calcium, creatinine, lactate dehydrogenase, magnesium, phosphate, potassium, sodium, total protein, uric acid. Can be performed +/- 3 days.
      9. Comprehensive Geriatric Assessment. Includes BMI and serum albumin.
      10. Note that any adverse event (AE) that meets the definition of a severe adverse event (SAE) must be reported from the signing of the main VICTOR trial informed consent form. All other AEs can be reported from the first dose of investigational medicinal product (IMP).
      11. Including number of days in hospital, days of intravenous antibiotic and antifungal treatment, number of blood components transfused.
      12. Patients who have resistant disease (those failing to achieve at least a partial response) after cycle 1, those with evidence of high risk disease by MRD assessment after cycle 2 (i.e. persistent MRD positivity in the peripheral blood in the daunorubicin, cytarabine and gemtuzumab ozogamicin (DAGO) arm), and those with confirmed molecular failure at any time should commence salvage chemotherapy and may be recommended for an allogeneic stem cell transplant (SCT).
      13. Gemtuzumab ozogamicin can be given on days 4 and 7 if white cell count ≥30 on day 1, provided the white cell count is <30 on day 4.
      14. VEN to be given on days 1-28 of each cycle, and LDAC on days 1-10. The subsequent cycle to be started upon count recovery and confirmation of disease status. After the fourth cycle of treatment, if the bone marrow aspirate shows continued morphological remission and tests negative for MRD (i.e., molecular complete response), patients will receive up to a further 8 cycles of VEN+LDAC up to a maximum 12 months of therapy. If the bone marrow aspirate at the end of cycle 12 tests shows ongoing molecular CR, patients will go on to receive a further one year (12 x 28 day cycles) of maintenance treatment with VEN.
      15. Cytarabine schedule varies according to age.
